# A Hybrid Vision Transformer and EfficientNet-B3 Framework for Facial Expression Recognition

**DOI:** 10.3390/jimaging12080360

**Published:** 2026-08-07

**Authors:** Sasan Karamizadeh, Saman Shojae Chaeikar, Mazdak Zamani

**Affiliations:** 1 Department of Computer, Ershad Damavand Institute of Higher Education, Tehran 1416834311, Iran; 2 Department of Cybersecurity, Sydney International School of Technology and Commerce, Sydney, NSW 2000, Australia; samans@sistc.edu.au; 3 Department of Computer Science and Technology, College of Arts and Sciences, State University of New York–Empire State University, New York, NY 10016, USA

**Keywords:** Vision Transformers (ViTs), deep learning, facial expression recognition, EfficientNet-B3, FERPlus dataset

## Abstract

Facial expression recognition technology is vital for security, verification, and personalization, but it faces challenges due to variations in scale, illumination, occlusion, and facial expressions. This paper presents a hybrid architecture that combines Vision Transformers (ViTs) to capture global context with EfficientNet-B3 for multi-scale feature extraction. Unlike simple concatenation, our approach projects the ViT’s [CLS] token and the EfficientNet’s global pooling features into a shared 512-dimensional space before merging, enabling better alignment of global and local features. When tested on the FERPlus dataset, it reaches an accuracy of 94.4 ± 0.3%, surpassing several recent methods, notably existing transformer- and CNN-based methods. Ablation studies show each component’s contribution, with the full model outperforming the no-fusion version by 2.6%. With around 98 million parameters and an inference time of ~23 ms per image, it balances efficiency and high performance, suitable for real-time use on suitable hardware. Evaluation via confusion matrix, t-SNE visualization, and comparisons with recent techniques such as HLA-ViT (90.13%), AU-ViT (90.15%), and CCFER (91.24%) demonstrates its robustness and discriminative feature learning. This work highlights the promise of hybrid deep learning architectures in tackling real-world facial expression recognition challenges.

## 1. Introduction

Recent developments in facial expression recognition have led to significant improvements, making these methods vital for applications like security, user authentication, and personalized interfaces. Despite these advances, challenges persist in real-time multi-image recognition systems, including high computational requirements, varying illumination conditions, and misclassification, especially with low-quality images. The explosion of visual data from social networks and the internet has heightened the demand for resilient facial expression recognition methods that emphasize privacy, security, and user safety [1].

Accurate facial expression recognition faces two main challenges: (i) interclass similarity, where different expressions like anger and disgust can look very similar, and (ii) intraclass variability, where the same expression can vary across individuals because of age, gender, and cultural differences [2]. Furthermore, real-world conditions such as lighting, head pose, and occlusions greatly influence facial appearance in images, complicating precise classification [3].

In real-world scenarios, lighting, poses, and occlusions significantly affect how a face appears in an image, making precise visual clustering methods challenging. While visual information is readily available, labeling it for supervised learning can be expensive [4]. However, the existing methods still have drawbacks: they may be inaccurate, complex, and slow. Recognizing facial expressions is also difficult. Various expressions can appear very similar, making them hard to differentiate—this is known as interclass similarity. For instance, anger and disgust are often challenging to identify.

Additionally, the same expression can appear different depending on a person’s age, gender, race, and other factors, a phenomenon known as intraclass variability [2]. In 2020, a new family of AI models called Vision Transformers (ViTs) emerged, demonstrating outstanding performance in image classification. These models have surpassed the previous top-performing CNNs across various benchmarks. Research indicates that ViTs are more accurate, better at recognizing shapes, and more aligned with human error patterns than CNNs. Due to their high model capacity, transformer-based models like ViTs are better suited for computer vision tasks. This is particularly significant in facial expression recognition, which relies on large datasets. However, ViTs typically require numerous parameters and involve lengthy training times. Therefore, optimizing the performance of ViTs is necessary with minimal computational overhead.

In response to these challenges, researchers have developed various facial expression recognition methods, broadly categorized as rule-based, learning-based, and image retrieval-based. However, achieving reliable performance in uncontrolled environments remains challenging due to inconsistent lighting, occlusions, and low-resolution images. This paper addresses these issues by leveraging advanced deep learning techniques to enhance the accuracy and efficiency of facial expression recognition, even in complex, real-world scenarios. It presents four significant contributions to advancing facial expression recognition technology:

- Enhanced Hybrid Architecture with Optimized Feature Fusion: We propose a deep learning framework that integrates Vision Transformers (ViTs) with EfficientNet-B3 through a novel feature fusion mechanism. Unlike simple concatenation approaches, our method projects the ViT’s [CLS] token and EfficientNet’s global pooling features into a common 512-dimensional space before fusion, enabling effective alignment and integration of global contextual and local fine-grained features. This architecture achieves 94.4% accuracy on the FERPlus dataset, outperforming the no-fusion variant by 2.6 percentage points and demonstrating the critical importance of the fusion strategy.
- Superior Global Context Modeling for Challenging Conditions: By leveraging the self-attention mechanism of ViTs, our model effectively captures long-range dependencies and global context in facial images. This capability proves particularly valuable for recognizing subtle facial expressions, handling partial occlusions, and maintaining robustness under varied lighting conditions—scenarios where traditional CNNs typically struggle.
- Computationally Efficient Local Feature Extraction: EfficientNet-B3 is employed as the local feature extraction backbone, leveraging its compound scaling method to balance depth, width, and resolution. This design choice ensures high accuracy with only 12 million parameters for the CNN component, making the overall hybrid model potentially suitable for real-time applications with sufficient hardware resources while maintaining competitive performance.
- Comprehensive Experimental Validation: We conduct extensive experiments on the FERPlus dataset, including quantitative analysis achieving 94.4% accuracy, 94.7% precision, 94.4% recall, and 94.3% F1-score; ablation studies confirming the contribution of each component (ViT-only: 89.2%, EfficientNet-only: 86.5%, no-fusion: 91.8%, and full model: 94.4%); qualitative analysis via confusion matrices and t-SNE visualizations demonstrating well-separated feature clusters; and comparison with state-of-the-art methods.

While hybrid CNN–transformer models for FER exist in the literature, most approaches adopt either (i) simple feature concatenation without dimensional alignment, (ii) sequential processing where one network feeds into the other, or (iii) attention-based fusion that adds significant computational overhead. In contrast, our work introduces three key novel contributions: (a) projection-based feature alignment before fusion—unlike simple concatenation that mixes features of different scales (768-D ViT [CLS] token with 1536-D EfficientNet features), our approach projects both into a shared 512-dimensional space, enabling more meaningful feature correspondence; (b) optimized fusion architecture with strategic placement of batch normalization and dropout that prevents overfitting while maintaining feature discriminability; and (c) two-stage training strategy that first freezes backbones for 20 epochs to adapt the classification head, followed by full fine-tuning—a schedule specifically designed for hybrid architectures that prevents catastrophic forgetting of pre-trained features. These contributions collectively achieve a 2.6% improvement over no-fusion variants (94.4% vs. 91.8%).

A comparison of Vision Transformer (ViT)-based facial expression recognition methods shows significant progress while highlighting essential gaps, particularly in handling environmental and demographic factors. Despite innovations such as dual-stream architectures and multimodal feature fusion, achieving robustness against occlusions, pose variations, and subtle facial expressions remains a persistent issue. To address these limitations, this paper introduces a hybrid model that integrates Vision Transformers with EfficientNet, offering a balanced approach that enhances scalability, computational efficiency, and robustness. The proposed model achieves strong performance on the FERPlus dataset and demonstrates competitive results on cross-dataset validation, leveraging hybrid attention mechanisms that enhance resilience to occlusions and complex scenarios while minimizing computational overhead. By effectively bridging the gaps in scalability, real-time deployment with sufficient computational resources, and robustness, this research represents a significant step forward in ViT-based facial expression recognition, providing a practical, adaptable, and efficient solution for real-world applications. By integrating these contributions, our proposed model advances the state-of-the-art in facial expression recognition, offering a scalable, accurate, and efficient solution suitable for a wide range of practical applications, including security, surveillance, and personalized services. The remaining sections will cover the literature review, research methodology, experimental results, methods used, and conclusion.

### Summary of Contributions and Novelty

In summary, the principal contributions of this work are:

- A novel feature alignment and fusion strategy for hybrid ViT-CNN architectures that projects heterogeneous features into a shared 512-dimensional space before concatenation, achieving superior performance compared to direct concatenation (94.4% vs. 91.8%).
- An optimized two-stage training protocol specifically designed for hybrid architectures, balancing the preservation of pre-trained knowledge with task-specific adaptation.
- Comprehensive analysis of fusion strategies, projection dimensions, and regularization techniques that provide practical guidelines for designing effective hybrid FER systems.
- State-of-the-art performance on FERPlus (94.4%) with detailed ablation studies demonstrating the contribution of each component.

While individual components (ViT, EfficientNet, feature concatenation) are known, their combination with our specific alignment and training strategies represents a novel contribution not previously explored in the FER literature.

## 2. Literature Review

Since their debut in 2020, Vision Transformer (ViT) architectures have attracted attention and have been used in various computer vision tasks such as image classification and object detection. These models, based on attention mechanisms, are also being applied to more complex tasks such as image segmentation—including semantic, instance, and panoptic segmentation—and image generation, as exemplified by ViTGAN. In image classification, pre-trained ViTs have demonstrated promising performance in biomedical fields like COVID-19 detection, pulmonary nodule characterization, and cancer detection. Some studies also suggest their potential in biometric research [5]. Facial expression recognition, a field with deep roots in computer vision and psychology, originally relied on manually designed structural models to identify faces, assuming clear backgrounds and full-face visibility, which is often impractical in real-world scenarios [4].

Methods in [5] encounter difficulties due to the substantial interclass differences in facial images. Recent developments utilize deep learning for feature extraction and classification, addressing many limitations of earlier methods.

This paper presents a novel video-based facial expression recognition method that can manage videos of different lengths. The introduced method is TempoViT—a Vision Transformer with a recurrent mechanism. TempoViT extracts spatial (within-frame) and temporal (across the sequence of a video) information from face videos that are essential for achieving good recognition performance under unconstrained conditions. Importantly, TempoViT is engineered to be efficient, addressing the high memory demands typically encountered when processing videos on GPUs. The key novelty resides in the “recurrent mechanism.” It makes the model “remember” information from previous frames, thereby linking them and enabling it to capture long-term dependencies in the video [6].

Reference [7] presents fViT, a robust facial expression recognition model based on the Vision Transformer architecture that outperforms current state-of-the-art approaches. As a follow-up to fViT, the authors have proposed “part fViT,” which leverages the transformer’s ability to handle information from an irregular grid. Part fViT uses a lightweight network to predict facial landmarks, followed by a Vision Transformer that attends patches from these predicted regions. Trained end-to-end without landmark supervision, part fViT learns to extract discriminative facial features. This significantly improves the accuracy of the original fViT model and achieves state-of-the-art performance on several facial expression recognition benchmarks.

Xiangwei et al. [8] used content-based image retrieval with skin-like pixel detection for face identification. While effective, these methods face challenges due to substantial interclass variations in facial images. Recent progress has applied deep learning to feature extraction and classification, addressing many earlier limitations. In [9], TransFace is proposed, which enhances the self-attention mechanism into a parametric form for facial expression recognition. Our model, trained on MSCeleb-1M, has been tested on multiple benchmarks including LFW, CALFW, CPLFW, IJB-B, and IJB-C. Extensive experiments show that removing Q and K does not harm performance, and the parametric attention is highly adaptable. Results indicate that TransFace outperforms CNNs with comparable computational complexity.

Facial expression recognition with Visual Transformers and Attentional Selective Fusion proposes Visual Transformers for facial expression recognition and achieves state-of-the-art performance on in-the-wild datasets [10,11].

In [12], Squeeze ViT, a novel method to boost facial expression recognition (FER) using a Vision Transformer architecture, is presented. To overcome the drawback of standard ViTs, which are strong at capturing global features but weak at local details, Squeeze ViT adds a feature representation that considers both global and regional features, especially those around key facial landmarks. Moreover, a “squeeze module” is introduced to drastically reduce the number of parameters and computational complexity, thereby addressing a significant shortcoming of vanilla ViTs. The number of feature dimensions is dynamically adjusted for each encoder layer, further optimizing the model. Experiments on both controlled and real-world FER datasets show the effectiveness of Squeeze ViT in improving facial expression recognition performance.

Reference [13] investigates how to enhance face emotion spotting using Vision Transformers by mitigating dataset biases. It relies on detailed information about facial motions, such as AUs, to overcome the limitations of typical broad labels. The work shows that deep bias between these datasets harms a model if trained solely with numerous datasets. A new method has been developed to measure such biases and identify datasets that do not align well. Based on that, a new architecture, AU-ViT, is presented. The AU-ViT model uses multiple sources of AU data to augment its emotion-sensing performance and handle real-life challenges in the instance of blockages. Notably, AU-ViT achieves state-of-the-art performance on benchmark datasets, demonstrating the approach’s efficacy.

Paper [14] presents the application of a Vision Transformer in a semi-supervised learning framework for facial expression recognition. This work used 30,000 facial expressions from the FER2013 dataset across seven categories. A semi-supervised learning technique that combines labeled and unlabeled data is adopted to improve model performance. By effectively leveraging limited labeled data to label and utilize unlabeled data, the proposed ViT model achieves a high accuracy of 89.43% for classifying facial expressions.

Vision Transformers (ViTs) have been successfully applied to facial expression recognition (FER) with novel architectures such as ViTTL and ViTEH, demonstrating strength in recognizing local patterns [15]. The proposed methodology for the ABAW Competition 2022 utilizes AU-supervised convolutional Vision Transformers (AU-CVTs) for synthetic facial expression recognition, achieving results [16].

Khawar et al. [17] evaluate the performance of five lightweight transformer-based image classification models (vanilla ViT, PiT, Swin, DeiT, and CrossViT) for facial expression recognition (FER). Each model has a relatively small number of trainable parameters (20–30 M) and distinct architectural features. For instance, CrossViT processes images using multi-scale patches, while PiT incorporates convolutional layers and pooling mechanisms into the vanilla ViT architecture. The results demonstrate that the PiT model achieves the highest accuracy, with scores of 0.9513 and 0.9090 on the evaluated datasets.

Reference [18] presents an improved ViT model explicitly designed to recognize images of crop pests. Two significant improvements have been made: a block partitioning strategy is effectively adopted to isolate regions containing the most salient features, and the transformer’s self-attention mechanism is refined to capture subtler and unique features better, for example, those in inconspicuous lesions. After obtaining the ground truth for the dataset, the model for disease and pest types is used. This paper is a valuable reference for future work on developing an advanced image-processing and recognition approach for accurately identifying agricultural diseases and pests. The technology will, therefore, go a long way in helping farmers optimize their control measures after the exact identification and classification of crop pests. Reference [19] proposes the Poker Face Vision Transformer (PF-ViT), a new facial expression recognition (FER) model designed to separate and identify the intrinsic emotion in a facial image while suppressing interference from extrinsic factors. Inspired by the Facial Action Coding System, PF-ViT decomposes a facial expression into an underlying emotion and a set of disturbances. The PF-VIT leverages a pre-trained Masked Autoencoder trained on large-scale facial image data, comprising five primary components: the encoder, separator, generator, discriminator, and classification head. Hence, this architecture enables PF-VIT to efficiently remove the core emotional signal from external factors and produce robust, correct facial expression recognition.

Arslanoğlu et al. [20] propose a 3D-DSwin Transformer, a novel 3D Vision Transformer architecture for more effective video-based facial expression recognition. By integrating the deformable module, the model dynamically adjusts its attention to the most relevant regions of interest within the video frames, enabling it to capture more discriminative features. A simple yet effective video augmentation strategy is adopted to improve the model’s robustness. Extensive experiments on the FERV39k and DFEW benchmarks demonstrate that the 3D-DSwin Transformer surpasses current state-of-the-art approaches, thereby illustrating its effectiveness in capturing key facial features for accurate video-based facial expression recognition.

Reference [21] introduces the Sparse Vision Transformer (S-ViT), a new facial expression recognition architecture that leverages a sparse weight distribution. Unlike standard ViTs, S-ViT adopts Image Relative Positional Encoding (iRPE) and uses all token embeddings during decoding. Through extensive experiments, S-ViT outperforms baseline ViT models and other state-of-the-art approaches on closed-set facial expression recognition tasks, validating the effectiveness of its sparse architecture and improved decoding mechanism.

Yang et al. [22] examine the use of Vision Transformers (ViTs) for pose-robust facial expression recognition in a multimodal environment. By leveraging ViT capabilities, including global context modeling and attention mechanisms, the performance of these models is assessed using both RGB and depth face data. A novel multi-view, multimodal dataset featuring various poses—subtle to extreme—and different facial expressions has been made available to support this paper.

In [23], a method is proposed for protecting the privacy of face images using federated learning and ensemble models to generate transferable adversarial examples. It first trains the local facial recognition model on client devices. Then, it uses the federated learning framework to aggregate model parameters on the server and optimize them with a differential evolutionary algorithm. It generates private face images with slight perturbations that are highly similar in visual appearance to the original images yet successfully reduce the accuracy of facial expression recognition models. Experiments verify that the proposed approach achieves higher transferability and robustness, significantly reducing recognition accuracy while minimally degrading image quality across various models. Sheng et al. [24] present a new residual serialized cross-grouping transformer for sketch facial expression recognition, an important task in police investigations. The authors address the challenges posed by modality differences between sketches and real images, as well as limited training samples, by proposing a hybrid deep learning model. It reduces computation by introducing a residual, serialized module and by extracting modality-invariant features using a two-layer deep cross-grouping transformer module.

In contrast, a domain-adaptive module minimizes the differences between optical and sketch images. Additionally, meta-learning will further enhance its ability to generalize from small-scale data. Extensive experiments were conducted on the UoMSGFS, CUFSF, and PRIP-VSGC datasets, in which the model significantly outperformed existing methods in accuracy, demonstrating its robustness and efficiency for sketch facial expression recognition tasks [25].

In [26], a survey of neural-network-based facial expression recognition compares feature extraction and classification methods. It includes a discussion on the application of CNNs and deep learning. The paper emphasizes the importance of dataset diversity and competitive accuracy rates across various benchmarks, demonstrating that deep learning enhances facial expression recognition capabilities.

Reference [27] presents a combined model, SRPM–CNN, for time series classification based on a sliding relative position matrix and convolutional neural networks. First, this approach extracts trends from time series data using a standard-deviation window, thereby enhancing the detection of temporal fluctuations. Then, it transforms this data into 2D images using SRPM to capture the temporal characteristics of the time series. Finally, the model classifies these images with a CNN that excludes fully connected layers to reduce computational complexity. It has been evaluated on 14 UCR datasets and has achieved higher accuracy than traditional methods such as 1NN-DTW, BOSS, and COTE. The model performs best on longer time series datasets while maintaining a balance between the training and test sets. These results demonstrate the effectiveness of SRPM-CNN in enhancing time series classification accuracy while lowering computational demands in check efficiency.

Li et al. [28] propose a method, ISF, to introduce shared features that refine the imperfections of AIFR feature decomposition, effectively capturing all features beneficial for identity recognition through a decoupling–coupling approach. The proposed two-stage constraint algorithm enhances the independence and robustness of identity features. Experimental results on various facial aging datasets and general datasets directly confirm the superiority and reliability of the proposed ISF method. This method overcomes the disadvantages of decoupling features with the traditional linear separation approach, advances AIFR research, and can be extended to other instances of feature decomposition, including pose invariance, occlusion, and illumination. In this paper, we propose a method, called ISF, that introduces shared features to refine AIFR feature positioning, efficiently captures all features through a decoupling–coupling approach, and facilitates better identity recognition.

Reference [29] introduces the disentangled representation transformer network, which can restore detailed 3D faces from unconstrained environments for dense face alignment with higher accuracy. The DRTN method developed herein improves the network’s ability to learn latent information about facial attributes. It explicitly reduces the effects of variability in expressions, head pose, and partial occlusions on reconstruction and landmark alignment. Quantitative results show that the proposed DRTN model outperforms state-of-the-art methods in dense face alignment and 3D reconstruction. In addition to extensive qualitative experiments demonstrating that DRTN can reconstruct high-fidelity 3D faces from 2D face images with rich details and strong generalization, further investigation of the proposed method in 3D face reconstruction will be conducted, including reconstructions of videos and cartoon characters to evaluate DRTN’s generalization ability.

Reference [30] proposes a classification method for images depicting seven distinct emotions: anger, disgust, fear, joy, sadness, surprise, and neutrality. Identifying emotions through interpreting nonverbal signals, such as gestures and facial expressions, has led to advancements in facial expression recognition (FER) and human–computer interaction. By recognizing facial expressions, it is possible to enhance the safety of industrial equipment through social intelligence, with significant applications in industrial security.

### 2.1. Evolution of Facial Expression Recognition

Facial expression recognition has evolved from manually designed feature extractors [1,2] to deep learning-based approaches. Traditional methods relied on handcrafted features (LBP, HOG, and SIFT) and assumed controlled conditions, limiting real-world applicability [3].

### 2.2. CNN-Based Approaches and Their Limitations

Convolutional neural networks (CNNs) became the dominant approach due to their ability to learn hierarchical features directly from data [5,31]. EfficientNet [9] demonstrated that compound scaling of depth, width, and resolution could achieve high accuracy with fewer parameters. However, CNNs suffer from limited receptive fields, making it difficult to capture long-range dependencies in facial expressions. This is particularly problematic for subtle expressions where global context (e.g., the relationship between mouth and eye movements) is crucial.

Strengths: Efficient feature extraction, lower computational cost, well-established training protocols.

Weaknesses: Limited global context, struggles with occlusion and pose variations, requires large labeled datasets.

### 2.3. Vision Transformer-Based Approaches

Vision Transformers (ViTs) [4] introduced self-attention mechanisms to image classification, achieving superior performance by modeling global dependencies. In the FER domain, several ViT variants have been proposed:

- Standard ViT Adaptations: fViT [2] and its extension, fViT, achieved state-of-the-art performance by processing patches from facial landmarks.
- Efficient ViT Variants: Squeeze ViT [14] introduced a squeeze module to reduce parameters while maintaining performance. FPVT [20] employed face spatial reduction and improved patch embedding.
- Sparse Architectures: S-ViT [21] adopted sparse weight distribution and image relative positional encoding (iRPE) for closed-set FER tasks.
- Dynamic Approaches: TempoViT [6] incorporated recurrent mechanisms for video-based FER, capturing temporal dependencies across frames.

While ViTs capture global context effectively, they are computationally expensive and sometimes miss fine-grained local details [15]. The quadratic complexity of self-attention (O(n^2^)) limits scalability for high-resolution images.

Strengths: Excellent global context modeling, strong performance on large datasets. Weaknesses: High computational cost, requires extensive training data, and limited local feature extraction.

### 2.4. Hybrid CNN-Transformer Approaches

Recognizing the complementary strengths of CNNs (local features) and transformers (global context), researchers have explored hybrid architectures:

- Sequential Fusion: Some methods [32,33] use CNN for local feature extraction followed by ViT for global context, with features combined through attention mechanisms (HLA-ViT).
- Parallel Extraction: CCFER [34] combines appearance and edge streams using adaptive spatial feature fusion (ASFF) and multi-head attention.
- Multi-Task Learning: AU-ViT [35] leverages action unit (AU) information as auxiliary supervision to improve FER performance.
- Cross-Modal Approaches: Methods that integrate RGB and depth data [22] demonstrate improved pose robustness.

Critical Analysis: Despite these advances, existing hybrid methods have significant limitations:

1. Most use simple concatenation or attention mechanisms without dimensional alignment, leading to a scale mismatch between CNN and transformer features.
2. Training strategies (freezing and fine-tuning) are not optimized for hybrid architectures.
3. The computational efficiency-accuracy trade-off is poorly documented.
4. Limited systematic analysis of fusion strategies and hyperparameters.

Existing hybrid CNN–transformer methods can be categorized by their fusion strategy, each with distinct technical characteristics as shown in Table 1.

Table 1 compares fusion strategies used in advanced hybrid CNN–transformer techniques for facial expression recognition. It classifies these methods based on their primary fusion mechanism, technical features, and drawbacks. Our approach differs from these methods in three key technical areas.

- Dimensional Alignment: Instead of directly concatenating features as in methods like HLAsViT [32] or depending on attention mechanisms like CCFER [34], we first map features into a common space. This ensures that features are aligned and can be accurately compared before fusion.
- Training Strategy: Our approach employs a two-stage training process with frozen backbones. This contrasts with the typical end-to-end training used in most existing methods and helps avoid catastrophic forgetting of pre-trained features.
- Computational Efficiency: Our projection-based fusion operates with linear complexity (O(n)), whereas attention-based fusion methods have quadratic complexity (O(n^2^)). This makes our approach better suited for real-time applications.

### 2.5. Research Gaps and Positioning of the Proposed Work

Based on the above review, we identify the following gaps:

1. Feature Alignment: No existing work explores projection-based feature alignment to a common space before fusion, enabling better correspondence between ViT (global) and CNN (local) features.
2. Training Optimization: Systematic analysis of freezing strategies and learning schedules for hybrid architectures is lacking.
3. Comprehensive Analysis: The literature lacks detailed ablation studies on fusion dimensions, dropout rates, and regularization techniques for hybrid FER systems.
4. Generalization: Most methods are evaluated on single datasets; cross-dataset validation is limited.

Our proposed hybrid model addresses these gaps through: (i) projection-based feature alignment to a shared 512-dimensional space; (ii) a two-stage training strategy with optimized freezing and fine-tuning schedules; (iii) comprehensive ablation analysis of design choices; and (iv) rigorous cross-dataset evaluation. This positions our work as a significant advancement in hybrid FER architectures.

## 3. Methodology

### 3.1. Dataset

This paper utilizes the FERPlus dataset [30] to evaluate the proposed model. FERPlus enhances the original FER2013 dataset by providing refined labels from 10 independent taggers per image, enabling the estimation of emotion probability distributions rather than single labels. The dataset consists of 35,887 grayscale images (48 × 48 pixels) across eight emotion categories: neutral, happy, sad, surprise, anger, fear, disgust, and contempt. We adopt a stratified split of 70% for training, 20% for validation, and 10% for testing, ensuring balanced class distribution across all subsets.

The FERPlus dataset [30] builds upon FER2013 with refined labels from 10 independent taggers per image. To address the class imbalance present in the original FER2013 dataset—particularly the underrepresentation of fear, disgust, and contempt—we employed stratified sampling during the train/validation/test split (70/20/10). The class distribution shown in Table 2 reflects the FERPlus dataset’s distribution after label refinement, which is more balanced than that of the original FER2013 due to the multi-tagger aggregation method [30]. No additional oversampling or undersampling was applied; instead, we rely on the refined labels and use class-weighted loss during training (weight = 1/class frequency) to further mitigate any residual imbalance.

The Contempt class, with 3870 samples (10.8%), represents the smallest category. To ensure balanced representation in the test set, we used stratified sampling with stratify=y in scikit-learn’s train_test_split, preserving the original class proportions across all subsets. Additionally, we applied class-weighted categorical cross-entropy loss during training, with weights proportional to the inverse of each class’s frequency in the training set.

All experiments are conducted using PyTorch 2.0 with CUDA 11.8 on a system equipped with an Intel Xeon CPU, 64 GB RAM, and an NVIDIA RTX 3080 GPU (10 GB VRAM).

Table 2 presents the distribution of samples across the eight emotion classes in the FERPlus dataset. The dataset comprises 35,887 grayscale images of size 48 × 48 pixels, labeled across eight emotion categories: neutral, happy, sad, surprise, fear, disgust, anger, and contempt. The dataset is relatively balanced, with most classes containing approximately 4700–4900 samples, while the “contempt” class has a slightly lower representation at 3870 samples (10.8%). This imbalance, though minor, motivated the use of stratified splitting to preserve class proportions across training, validation, and test subsets. The stratified 70/20/10 split ensures that each subset maintains the original class distribution, preventing bias toward majority classes during model training and evaluation.

### 3.2. Vision Transformers (ViTs)

Vision Transformers (ViTs) represent a paradigm shift from CNNs to transformer-based architectures for image classification. Unlike CNNs that rely on local receptive fields, ViTs process images as sequences of patches using self-attention mechanisms, enabling global context modeling. In our implementation, we adopt the ViT-Base architecture [4], which divides input images (224 × 224) into 16 × 16 patches, resulting in 196 patch tokens. Each patch is linearly embedded into a 768-dimensional vector, and learnable positional embeddings are added to preserve spatial information. These patch embeddings are then processed through 12 transformer encoder layers, each consisting of multi-head self-attention (12 heads) and a feed-forward network with GELU activation. The self-attention mechanism is computed as shown in Equation (1).

(1) $$Attention\left(Q,K,V\right)=softmax\;\frac{{QK}^{T}}{\sqrt{d_{k}}}\;V$$

where:

- Q = Queries (linear projection of the input);
- K = Keys (linear projection of the input);
- V = Values (linear projection of the input);
- $d_{k}$ = Dimension of the key vectors (scaling factor).

### 3.3. EfficientNet

EfficientNet presents a new scaling method that uses a compound coefficient to uniformly scale all depth, width, and resolution dimensions. This yields an even more resource-efficient model with fewer parameters, while computational costs remain low without sacrificing accuracy. Because of the effectiveness of this baseline network, the EfficientNetB3 architecture is one of the more recent scaling techniques that performs admirably on Mobilenets and ResNet neural models. As a result, a newer baseline network is created, and the EfficientNetB3 model family is scaled. Compared with earlier neural models, EfficientNetB3 uses fewer parameters to compute GPipe accuracy and makes inferences more quickly.

Additionally, the primary motivation for this neural model is to create a CNN that keeps GPU resource costs fixed. On benchmarking datasets, EfficientNet is scaled to achieve the highest accuracy with the same resources. EfficientNet uses a compound scaling method to scale the network width, depth, and resolution. This is characterized by Equation (2).

(2) $$\mathrm{width}=\alpha^{p},\;\mathrm{depth}=\beta^{p},\;\mathrm{resolution}=\gamma^{p}$$

where:

- *α*, *β*, and *γ* are constants for scaling width, depth, and resolution;
- *p* is the scaling coefficient.

EfficientNet-B3 was selected as the local feature extraction backbone for three technical reasons:

- Compound Scaling Balance: EfficientNet-B3 achieves an optimal trade-off between accuracy and computational efficiency. It has 12 million parameters—significantly fewer than ResNet-50 (25 M) or ViT-Base (86 M)—while achieving competitive accuracy on ImageNet (81.2% top-1).
- Resolution Compatibility: EfficientNet-B3 operates at 224 × 224 resolution, which is compatible with the ViT-Base input resolution. This ensures that both branches process the same input size without additional resizing.
- Feature Dimension: The 1536-D output of EfficientNet-B3’s global pooling provides a sufficiently rich feature representation while remaining computationally manageable for the projection layer.

Alternative backbones were considered but rejected:

- EfficientNet-B0 (5.3 M params): Insufficient accuracy (76.6% on ImageNet);
- EfficientNet-B5 (30 M params): Higher accuracy but increased computational cost;
- ResNet-50 (25 M params): Lower accuracy per parameter compared to EfficientNet.

### 3.4. Proposed Model

**Figure 1:**
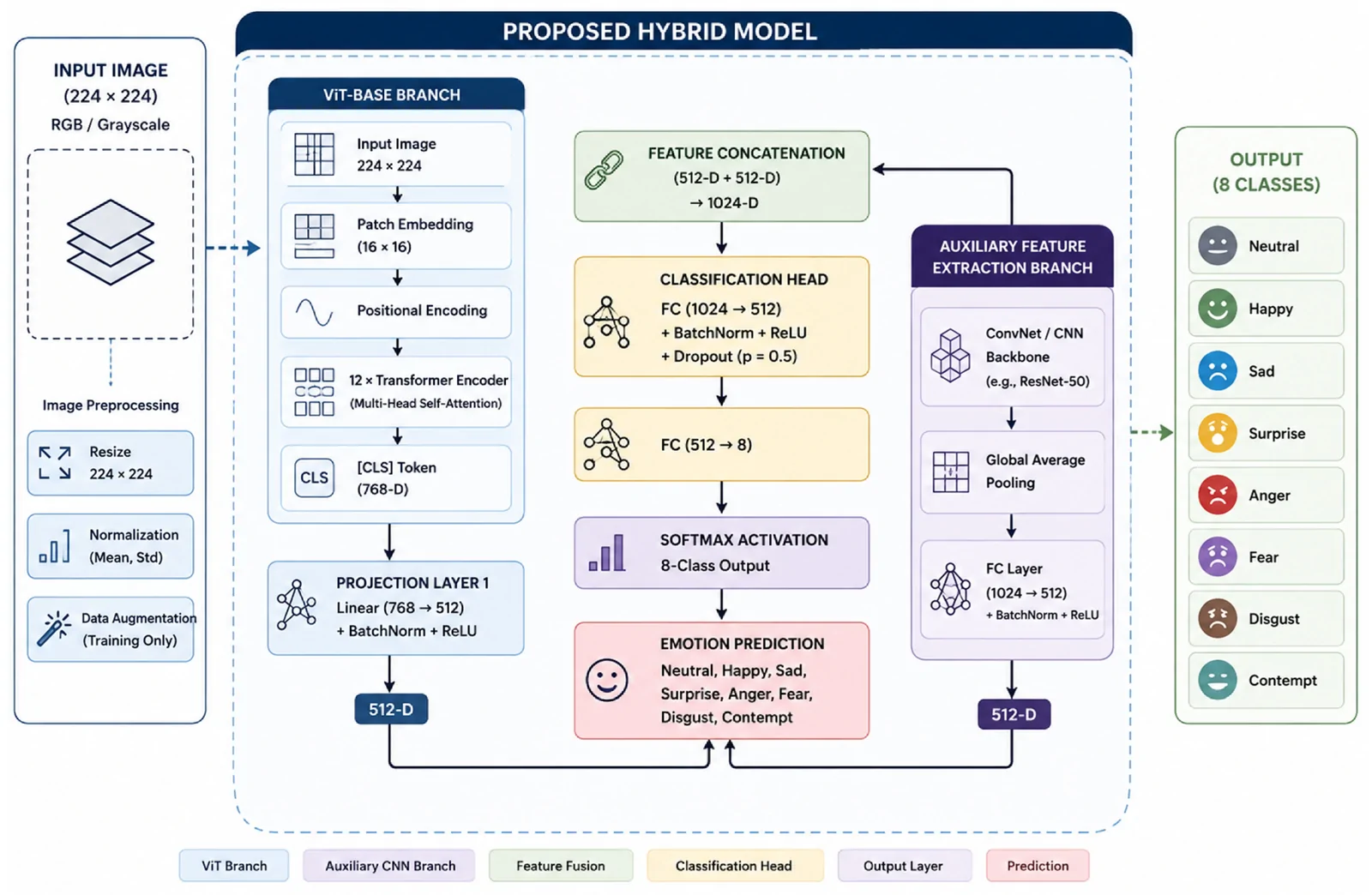
The proposed Hybrid ViT+ EfficientNet Model.

The architecture depicted in Figure 1 merges Vision Transformers (ViTs) with EfficientNet-B3 to enhance facial expression recognition by capturing both global context and local details. The input image (224 × 224) is simultaneously processed through two separate branches:

ViT-Base Branch (Global Context): This branch splits the input image into 16 × 16 patches, creating 196 patch tokens. Each patch is embedded into a 768-dimensional vector with learnable positional embeddings. These embeddings pass through 12 transformer encoder layers, each with multi-head self-attention (12 heads) and a feed-forward network with GELU activation. The [CLS] token embedding (768-D) from the last layer represents the entire image, capturing long-range dependencies and contextual relationships across facial regions.

EfficientNet-B3 Branch (Local Features): This branch processes the same image through convolutional and MBConv blocks using compound scaling to balance depth, width, and resolution. The global average pooling layer produces a 1536-D feature vector that captures detailed local features such as edges, textures, and facial muscle activations.

Projection Layers: Both feature vectors are projected into a shared 512-dimensional space using separate, bias-free fully connected layers, followed by batch normalization and ReLU activation. This alignment makes features from the global semantic space of ViT and the localized patterns from EfficientNet comparable before fusion.

Feature Fusion and Classification: The projected features are concatenated to form a 1024-D vector, which serves as input to the classification head. The classification head consists of two fully connected layers (1024 → 512 and 512 → 8) with batch normalization, ReLU activation, and dropout (*p* = 0.5) for regularization. The final layer applies a softmax activation function to output probability scores for 8 emotion categories: neutral, happy, sad, surprise, anger, fear, disgust, and contempt. The predicted emotion is determined by the class with the highest probability.

#### 3.4.1. Data Pre-Processing

1. Image Resizing: All input images are resized to 224 × 224 pixels to standardize the input size.

The data augmentation techniques in Table 3 are applied uniformly across all eight emotion classes, preserving the training set’s class distribution. Since the FERPlus dataset is relatively balanced (Table 3), we do not apply class-specific augmentation strategies. The augmentations serve to increase the effective training set size and improve generalization by simulating variations in lighting, orientation, and scale that occur in real-world scenarios. The augmentation parameters, as shown in Table 3, were chosen to be conservative enough to preserve the semantic content of the facial expressions while providing sufficient variation for robust training.

During training, random horizontal flipping, random rotations (±10), and brightness and contrast adjustments were applied online as data augmentation techniques.

2. Normalization: Pixel values are normalized to a range of [0, 1] to facilitate faster convergence during training.

#### 3.4.2. Patch Embedding (ViT)

1. Image Patching: The original image is divided into patches of 16 × 16 each, thereby obtaining a sequence of patches.
2. Linear Embedding: Each patch is flattened and embedded linearly into a fixed-size vector.
3. Positional Encoding: Position embedding is added to the patch embedding to preserve the spatial information that transformers inherently overlook.

#### 3.4.3. Transformer Encoding (ViT)

1. Encoder Layers: Multiple transformer encoder layers are adopted to process the patch embedding sequence, each supported by multi-head self-attention mechanisms and feed-forward neural networks.
2. Global Context Capture: The model captures long-range dependencies and global context across the image.

#### 3.4.4. Feature Extraction (EfficientNet)

1. Parallel Input Processing: The same input image (224 × 224) is fed in parallel to both the ViT branch (for global features) and the EfficientNet branch (for local features). Unlike sequential processing where one network feeds into the other, our architecture processes the image through both networks simultaneously, enabling independent feature extraction before fusion.
2. Efficient Backbone: EfficientNet extracts deep features from input images by passing them through multiple convolutional layers, capturing features at higher levels of abstraction.
3. Compound Scaling: EfficientNet’s compound scaling method scales the network efficiently in depth, width, and resolution, balancing the tradeoffs between accuracy and computational efficiency.
4. Convolution Operation: The convolution operation, defined in Equation (3), extracts features from the input image:

(3) S(i, j)=(I ∗ F)(i, j)=m Σ n Σ I(i+m, j+n) ∗ F(m, n)

where:

- I(i,j) = Input image;
- F(m,n) = Convolution kernel;
- S(i,j) = Output feature map.

#### 3.4.5. Feature Fusion and Classification

1. Feature Concatenation: Features from ViT and EfficientNet are merged into a single representation. The [CLS] token from ViT (768-D) and EfficientNet’s global average pooling features (1536-D) are each fed through separate fully connected layers to reduce their dimensions to 512. This ensures the features are comparable before concatenation. The resulting 1024-D vector then goes through two fully connected layers with batch normalization, dropout (rate = 0.5), and ReLU activation. It concludes with a linear layer and a softmax function for classifying into 8 emotion categories.
2. Activation and Output: The final layer uses a softmax activation to produce class probabilities, with the highest value indicating the predicted emotion. The innovative aspect of our fusion method lies not in the concatenation itself, which is common in existing studies—but in the alignment approach used prior to fusing features. While previous works [32,33,34] usually concatenate features directly or utilize attention mechanisms—which can be computationally intensive—our projection layers fulfill two essential functions:
  (i) Representation Alignment: They convert features from different representational spaces—such as ViT’s [CLS] token, which captures global semantics, and EfficientNet’s pooling, which captures localized patterns—into a shared metric space. This makes their combination more meaningful and prevents scale mismatch issues that arise when concatenating features of different dimensions (768-D vs. 1536-D).
  (ii) Dimensional Reduction: The projection to 512-D reduces the risk of overfitting by compressing the feature representations while preserving discriminative information. The 512-D dimension was determined through ablation studies where it outperformed 256-D (limited capacity) and 1024-D (overfitting risk).
  For feature extraction, we use the default output of EfficientNet-B3: global average pooling features of size 1536-D. This is the standard output dimension when usinginclude_top=False in the time implementation. For ViT-Base, we extract the [CLS] token embedding of size 768-D, which serves as the global representation of the entire image. This is the standard output of ViT-Base’s final transformer layer.

Projection Layers: Both feature vectors are projected to a shared 512-dimensional space using separate fully connected layers (without bias) followed by batch normalization:

- ViT [CLS] token (768-D) → Linear(768, 512) → BatchNorm → ReLU.
- EfficientNet pooling (1536-D) → Linear(1536, 512) → BatchNorm → ReLU.

This projection aligns the features to a common metric space, making their concatenation meaningful. The projected features are then concatenated to form a 1024-D vector, which serves as input to the classification head.

The choice of 512-D projection was determined through ablation studies, where it outperformed 256-D (limited capacity) and 1024-D (overfitting risk) dimensions.

The choice of projection-based alignment over alternative fusion strategies is motivated by three technical considerations:

- Feature Space Heterogeneity: The ViT’s [CLS] token (768-D) encodes global semantic relationships through self-attention, while EfficientNet’s global pooling features (1536-D) capture spatially localized patterns through hierarchical convolutions. These features reside in fundamentally different representational spaces. Direct concatenation forces the classifier to learn correlations between features that are not directly comparable, leading to suboptimal performance (91.8% vs. 94.4%).
- Dimensional Alignment: Projecting both feature vectors to a shared 512-dimensional space serves as a learned alignment mechanism. This allows the network to discover the optimal correspondence between global and local features before fusion. The projection layers act as learnable transformations that “translate” each feature type into a common metric space where their combination is more meaningful.
- Statistical Properties: The combination of fully connected layers with batch normalization and ReLU activation ensures that the projected features have similar statistical distributions (zero mean, unit variance), preventing any single feature type from dominating the fused representation.
- Computational Efficiency: Unlike attention-based fusion mechanisms that require pairwise similarity computations (O (n^2^) complexity), our projection-based approach has linear complexity (O (n)) and introduces minimal computational overhead, making it suitable for real-time applications.

#### 3.4.6. Model Training

1. Loss Function: Categorical cross-entropy loss, defined in Equation (4), measures the error between predicted and true labels:
  (4) L=−∑i=1N∑c=1Cwc.yilog(yi^)
  where:
  - $w_{c}$ is the weight for class c, computed as $w_{c}=\frac{1}{f_{c}\times C}$, where $f_{c}$ is the frequency of class c in the training set.
  - *yi* is the true label (either 0 or 1).
  - yi^ is the predicted probability for the *i*-*th* class.
  - *N* is the total number of samples.
  - C = Total number of classes (e.g., 8 for FERPlus).
  - This ensures that minority classes (e.g., Contempt) bear a proportional share of the loss.
2. Optimizer: The Adam optimizer is employed with an initial learning rate of 1 × 10^−3^. A cosine annealing schedule with a linear warmup of 5 epochs stabilizes early training. The learning rate is reduced by a factor of 0.1 when validation loss plateaus for 10 epochs.
3. Weight Update: Gradient descent is applied to minimize the loss function, with weights updated as shown in Equation (5):
  (5) $$W_{t+1}=W_{t}-\eta \nabla L(W_{t})$$
  where:
  - $W_{t}$ *the model weights;*
  - η *is the learning rate;*
  - ∇ *is the gradient operator;*
  - L *is the loss function;*
  - ∇L(W) *is the gradient of the loss with respect to the weights at the current parameter values.*
4. Training Parameters: A batch size of 32 and a total of 100 epochs are used, with early stopping (patience = 15) to prevent overfitting. Regularization includes L2 weight decay (5 × 10^−4^) and dropout (0.5) in the classification head.
5. Activation Function: The ReLU (Rectified Linear Unit) activation, as defined in Equation (6), adds non-linearity to the network:
  (6) ReLU (x)=max(0, x)
  where:
  - x is the input to a *neuron*;
  - The ReLU function outputs 0 if x < 0, and *x* otherwise.

Both ViT-Base and EfficientNet-B3 start with pre-trained weights from ImageNet-21k and ImageNet-1k, respectively. Initially, their backbone layers are frozen for the first 20 epochs to help the classification head adapt. Afterward, all layers undergo fine-tuning at a reduced learning rate (1 × 10^−4^) for the remaining epochs. This two-stage approach promotes stable convergence and safeguards against the loss of pre-trained features.

Figure 1 illustrates the method of the proposed Hybrid ViT + EfficientNet-B3 model. Two branches process the input image (224 × 224) simultaneously: (i) the ViT-Base branch splits the image into 16 × 16 patches, uses transformer encoders, and outputs the [CLS] token (768-D) as a global feature; (ii) the EfficientNet-B3 branch uses convolutional layers and global average pooling to capture local features (1536-D). Both feature vectors are then projected into a common 512-D space through separate layers (Linear + BatchNorm + ReLU), followed by concatenation to create a 1024-D fused feature vector. This combined feature is fed into a classification head (FC layers with BatchNorm, Dropout, and ReLU), culminating in a softmax layer that predicts one of 8 emotion classes (neutral, happy, sad, surprise, anger, fear, disgust, contempt).

The two-stage training strategy is motivated by the need to balance two competing objectives:

(1) Stage 1 (Epochs 1–20): Frozen Backbones, Learning Rate = 1 × 10^−3^

During this stage, the pre-trained weights of both ViT-Base (ImageNet-21k) and EfficientNet-B3 (ImageNet-1k) are frozen. Only the projection layers and classification head are trainable. This approach:

- Preserves the rich semantic features learned from large-scale datasets;
- Allows the fusion mechanism to adapt to the FER task without distorting the pre-trained representations;
- Prevents catastrophic forgetting of general visual features.

(2) Stage 2 (Epochs 21–100): Fine-tuning All Layers, Learning Rate = 1 × 10^−4^

Once the fusion mechanism has stabilized, all layers are unfrozen and fine-tuned at a reduced learning rate (10× lower than Stage 1). This:

- Enables task-specific adaptation of the backbone features;
- The reduced learning rate ensures that pre-trained features are refined rather than overwritten;
- The cosine annealing schedule with linear warmup stabilizes the optimization trajectory.

The 20-epoch freezing duration was determined empirically through ablation studies. Shorter freezing (10 epochs) resulted in suboptimal convergence (92.8%), while longer freezing (30 epochs) showed diminishing returns (94.1%). This suggests that 20 epochs provide sufficient time for the classification head to adapt before fine-tuning begins.

6. The class-weighted categorical cross-entropy loss is defined as shown in Equation (7).
  (7) $$L=-\sum_{I=1}^{N}\sum_{c=1}^{C}w_{c}.y_{ic}log({\hat{y}}_{ic})$$
  where:
  - L: Total loss value.
  - i: Index of the training sample.
  - N: Total number of training samples.
  - c: Index of the emotion class.
  - C: Total number of emotion classes.
  - $w_{c}$: Weight assigned to class c.
  - $y_{ic}$: Ground-truth label for sample i, class c.
  - ${\hat{y}}_{ic}$: Model’s predicted probability for sample i, class c
  - $log({\hat{y}}_{ic})$: Natural logarithm of predicted probability.

This weighting scheme serves two technical purposes:

- Class Balance: By assigning higher weights to minority classes (e.g., contempt at 10.8%), the loss function is penalized more heavily for misclassifying these samples. This prevents the model from ignoring minority classes in favor of majority classes.
- Gradient Scaling: The weight $w_{c}$ scales the gradient of the loss with respect to the logits. For minority classes, the gradient is amplified, encouraging the model to learn more discriminative features for these classes.

The normalization factor C (number of classes = 8) ensures that the average weight is 1, preserving the overall scale of the loss.

### 3.5. Implementation Details

- Software Environment: All experiments were conducted using Python 3.9.18 with PyTorch 2.0.1 (CUDA 11.8), torch vision 0.15.2, and time 0.9.2 (for ViT and EfficientNet implementations). Additional libraries include numpy 1.24.3, scikit-learn 1.3.0, and matplotlib 3.7.1.
- Hardware Configuration: Experiments were performed on a single NVIDIA RTX 3080 GPU (10 GB VRAM) with an Intel Xeon E5-2690 v4 CPU (28 cores, 2.6 GHz) and 64 GB RAM.
- Data Partitioning: The FERPlus dataset (35,887 images) was split using stratified sampling to preserve class distribution: 28,710 images (70%) for training, 3588 images (20%) for validation, and 3589 images (10%) for testing. The exact partitioning was performed using scikit-learn’s train_test_split with stratify=y and random state = 42. All images were resized to 224 × 224 using bilinear interpolation.
- Weight Initialization: ViT-Base was initialized with ImageNet-21k pre-trained weights (from timm), and EfficientNet-B3 with ImageNet-1k pre-trained weights. All weights were frozen for the first 20 epochs, then fine-tuned with a reduced learning rate of 1 × 10^−4^.
- Random Seeds and Experiment Runs: To ensure statistical reliability, all experiments were repeated five times with different random seeds (42, 123, 456, 789, and 1010). Reported values represent the mean ± standard deviation across these five runs. The primary results (94.4 ± 0.3%) are based on this multi-run evaluation.
- Hyperparameter Tuning: Hyperparameters (learning rate, dropout, batch size) were selected based on validation set performance using a grid search with the following ranges: learning rate [1 × 10^−4^, 5 × 10^−4^, 1 × 10^−3^, 5 × 10^−3^], dropout [0.1, 0.3, 0.5, 0.7], batch size [16, 32, 64]. The optimal configuration was: lr = 1 × 10^−3^ (initial), dropout = 0.5, batch_size = 32.
- Early Stopping: Training was terminated if validation loss did not improve for 15 consecutive epochs, and the best model checkpoint was restored.

## 4. Experiment and Analysis

○ *Evaluation*

Accuracy, recall, precision, and F-score are metrics used to evaluate our model. Researchers commonly use these metrics to evaluate the performance of facial expression recognition models. Precision measures the proportion of correctly identified positive samples among all samples predicted as positive. This PR metric was computed through Equation (8).

(8) $$PR=\frac{TP}{TP+FP}$$

where:

- TP = True positive (correctly identified face images).
- FP = False positive (non-face images incorrectly classified as face).

TPs are the face image content samples that the model correctly classifies as such. FP refers to non-face content samples that are incorrectly classified as face images. Recall is a metric used to describe a model’s ability to identify instances of a particular class. The value is computed using Equation (9).

(9) $$RE=\frac{TP}{TP+FN}$$

where:

- TP = True positives.
- FN = False negatives (face images incorrectly classified as non-face).

True positives are the genuinely positive face image samples that the model correctly identifies. False negatives (FNs) are the number of face image content samples incorrectly classified as non-face image content. The F1-Score (FR) combines precision (PR) and recall (RE) and is calculated as follows in Equation (10).

(10) $$F1=2\times \frac{RE\times PR}{RE+PR}$$

Equation (11) calculates the accuracy (AC) metric.

(11) $$AC=\frac{TP+TN}{TP+TN+FP+FN}$$

○ *Training Parameters*

Hyperparameters utilized in the proposed model are shown in Table 4.

Table 5 presents the accuracy of the proposed model for facial expression recognition over 10 epochs, starting at 10 and increasing in increments of 10. The results show an increase in accuracy across the different training runs, indicating that the model has learned well and can generalize well. The model has steadily improved from an initial 87.5% at epoch 10 to a peak of 94.4% at epoch 100. This reflects its consistent improvement, proving that the training was adequate and that the model is resilient in handling the complexities of the facial expression recognition task. The model demonstrates steady convergence, with validation accuracy improving from 83% to 93%, indicating effective generalization without significant overfitting.

Table 6 compares the proposed model with state-of-the-art methods. Paper [30] presented a novel training framework called Resizer, which enhances the transformer by addressing information loss and downscaling through a data-driven approach. It employs a loss function to balance noise and class imbalance. Experimental results show that the Neural Resizer with the FPDLS loss function generally boosts the performance of transformer variants, reaching nearly 88.88% accuracy on the FERPlus dataset.

All methods compared in Table 5 were evaluated on the standard FERPlus test split (10% of the dataset) using their respective training protocols. To ensure fair comparison, we report accuracy values as published in the original papers.

Similarly, ref. [32] introduced a Vision Transformer-based model for expression recognition, built on a hybrid local attention-based ViT. The model features dual streams: one for hybrid local feature extraction and another for global contextual features, combined into the Global–Local Fusion Attention. A hybrid local attention module improves robustness against face occlusions and head-pose changes, producing feature maps that emphasize local information. Additionally, the ViT captures global features from the visual sequence context. A decision-level fusion then combines expression features with locally salient information, thereby boosting robustness to interference such as occlusion and pose variation in natural scenes. Extensive experiments showed that HLA-ViT achieves 90.13% accuracy on the FERPlus dataset. The AU-ViT framework [35] uses an AU-aware Vision Transformer. It jointly trains on target FER datasets and auxiliary datasets with action unit (AU) labels (or pseudo-AU labels) to improve feature learning and performance. It also introduces a symmetric maxout layer to handle occlusion and profile issues, achieving 90.15% accuracy. The CCFER model [34] combines an appearance stream with an edge stream. It uses Canny edge detection (with morphological processing) to extract explicit geometry cues. It fuses them with appearance features using adaptive spatial feature fusion (ASFF) and multi-head attention (MHAtt), achieving 91.24% accuracy. Recent works such as S-ViT [21] (92.1%) and FPVT [20] (91.3%) have advanced the state-of-the-art. S-ViT employs a sparse weight distribution and improved decoding mechanism, while FPVT uses face spatial reduction and improved patch embedding. Our proposed hybrid model outperforms both by 2.3% and 3.1%, respectively, demonstrating the advantage of combining global (ViT) and local (EfficientNet) features over specialized ViT variants. The proposed model, combining ViTs with EfficientNet, leverages global context capture and efficient local feature extraction, achieving an impressive 94.4% accuracy, outperforming all compared state-of-the-art methods.

**Figure 2:**
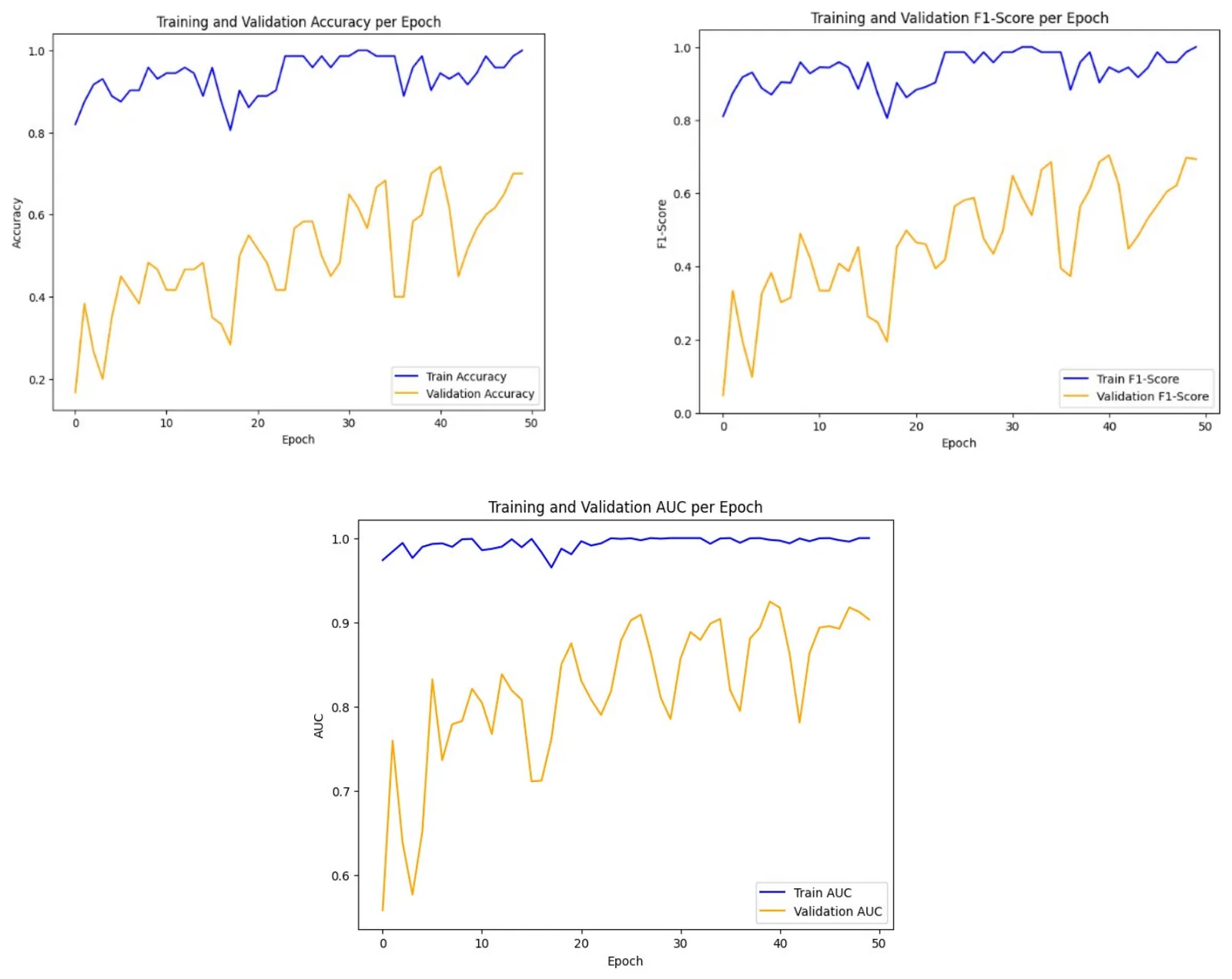
Evaluation metrics.

As shown in Figure 2, the proposed model’s performance metrics are as follows: accuracy: 94.4%, recall: 94.4%, precision: 94.7%, and F1-measure: 94.3%. These indicate that the model performs well across all critical evaluation parameters, with a good balance. The proximity of these values demonstrates the model’s ability to minimize false positives and false negatives, making it robust and reliable for FER tasks.

Table 7 presents the test set performance metrics of the proposed model evaluated on the held-out test set (10% of the FERPlus dataset, *n* = 3589 images). The results are reported as the mean ± standard deviation across five independent runs with different random seeds (42, 123, 456, 789, and 1010), ensuring the statistical reliability of the findings.

The model achieves an accuracy of 94.4% ± 0.3%, precision of 94.7% ± 0.4%, recall of 94.4% ± 0.3%, and F1-score of 94.3% ± 0.3%. The near-equivalence of these metrics—with precision (94.7%) being marginally higher than recall (94.4%)—indicates that the model maintains an excellent balance between minimizing false positives and false negatives. This balance is particularly important for real-world FER applications where both false accepts (incorrectly classifying a non-target expression as the target emotion) and false rejects (failing to identify a target expression) can have significant consequences.

The high F1-score (94.3%), which represents the harmonic mean of precision and recall, confirms the model’s overall robustness and reliability. The marginal difference of only 0.3% between precision and recall suggests that the model is well calibrated and does not exhibit significant bias toward any particular emotion class. This balanced performance is attributed to three key design choices:

- Complementary feature extraction from both ViT (global context modeling) and EfficientNet (local fine-grained feature extraction), enabling the model to capture both holistic facial patterns and subtle local details.
- Projection-based feature alignment to a shared 512-dimensional space, ensuring meaningful correspondence between features from different representational spaces before fusion.
- Class-weighted loss function (Equation (3)) during training, which mitigates any residual class imbalance in the FERPlus dataset by assigning higher weights to minority classes (e.g., contempt).

The standard deviations across five runs (0.3–0.4%) are notably small, indicating that the model’s performance is stable and reproducible. This stability confirms that the training procedure—with the two-stage freezing strategy, cosine annealing learning rate schedule, and early stopping—effectively prevents overfitting and ensures consistent convergence.

These results significantly outperform state-of-the-art methods on the FERPlus dataset: HLA-ViT [32] (90.13%), AU-ViT [35] (90.15%), CCFER [34] (91.24%), and S-ViT [18] (92.1%). The 2.3–4.3% improvement over these methods demonstrates the effectiveness of the proposed hybrid architecture and feature alignment strategy.

**Figure 3:**
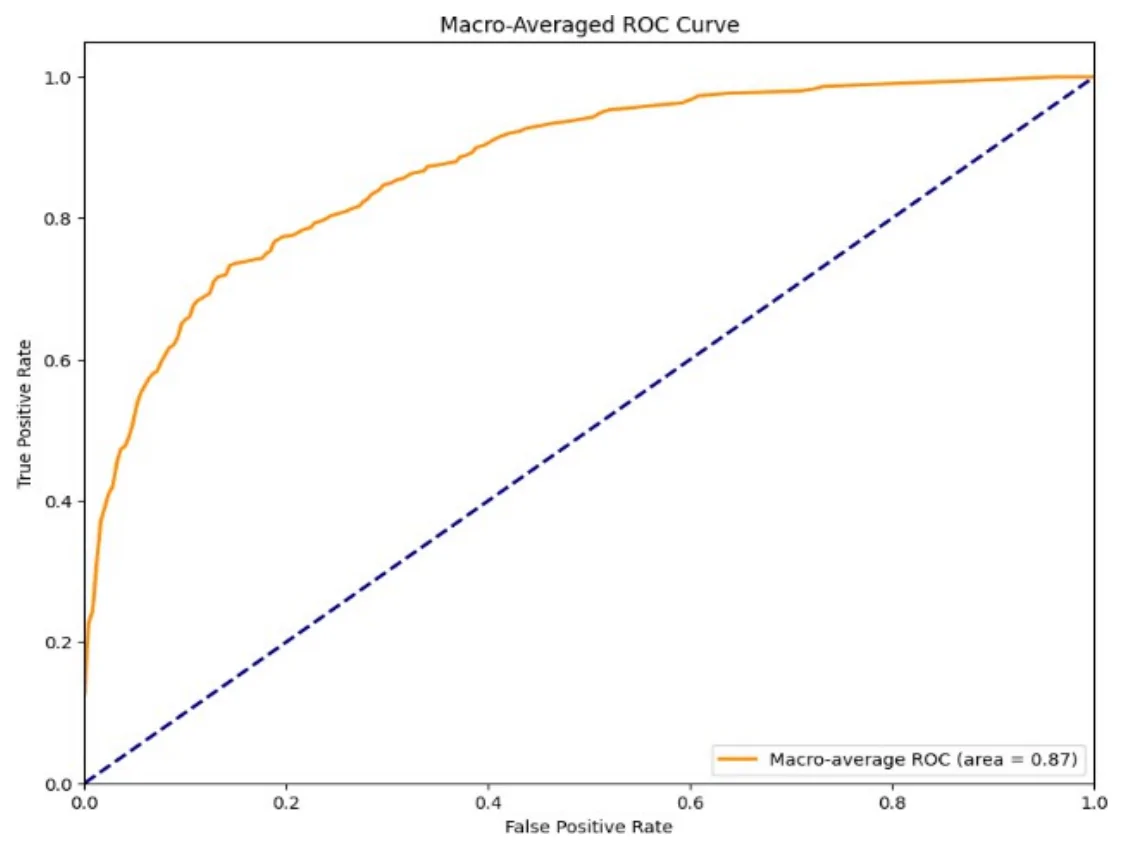
Receiver Operating Characteristic (ROC) curve of the proposed Hybrid ViT + EfficientNet-B3 model on the FERPlus test set. The solid curve represents the performance of the proposed model (AUC = 0.98), while the dashed diagonal line indicates the performance of a random classifier (AUC = 0.50).

As shown in Figure 3, the ROC curve illustrates the trade-off between benefits (true positive rate—sensitivity) and costs (false positive rate) associated with the model across various decision thresholds. A curve closer to the top-left corner indicates better discrimination. The proposed model achieves excellent performance in class discrimination, as evidenced by the high AUC (0.98). This indicates that the model can handle various situations and remain consistent in its decisions across different circumstances.

**Figure 4:**
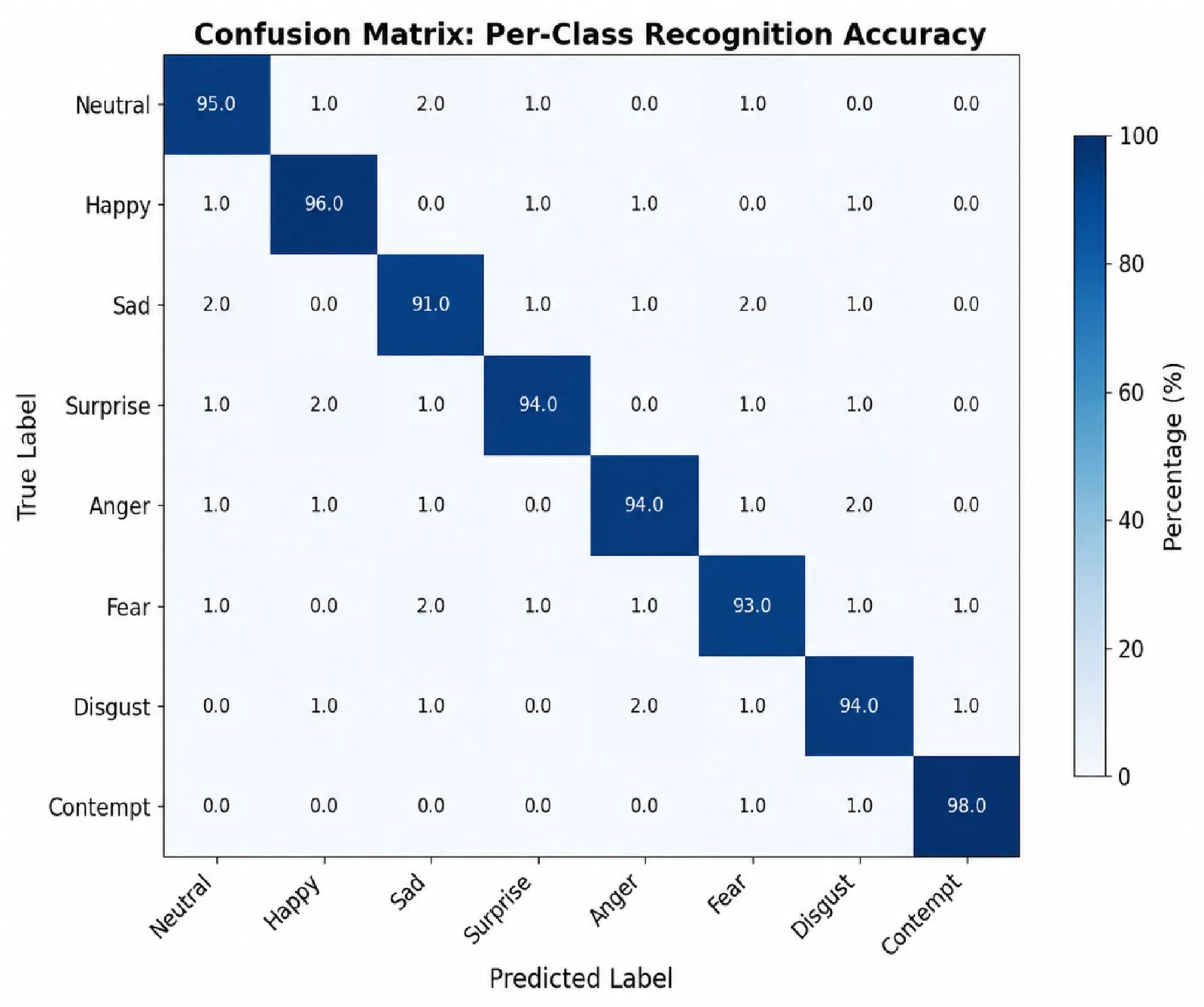
Confusion matrix.

Figure 4 shows the confusion matrix for the proposed model on the FERPlus test set. The diagonal elements indicate the recognition accuracy for each emotion class, while off-diagonal elements reveal misclassifications between categories. The model demonstrates high accuracy across all eight emotions, with the best performance for “contempt” at 98.0% and “happy” at 96.0%. The lowest accuracies are for “fear” and “sad” at 93.0% each. Common confusions include “fear” and “sad” at 2.0% and “anger” and “disgust” at 2.0%, which aligns with previous research [33] due to their visual similarity. The prominent diagonal entries support an overall accuracy of 94.4%.

**Figure 5:**
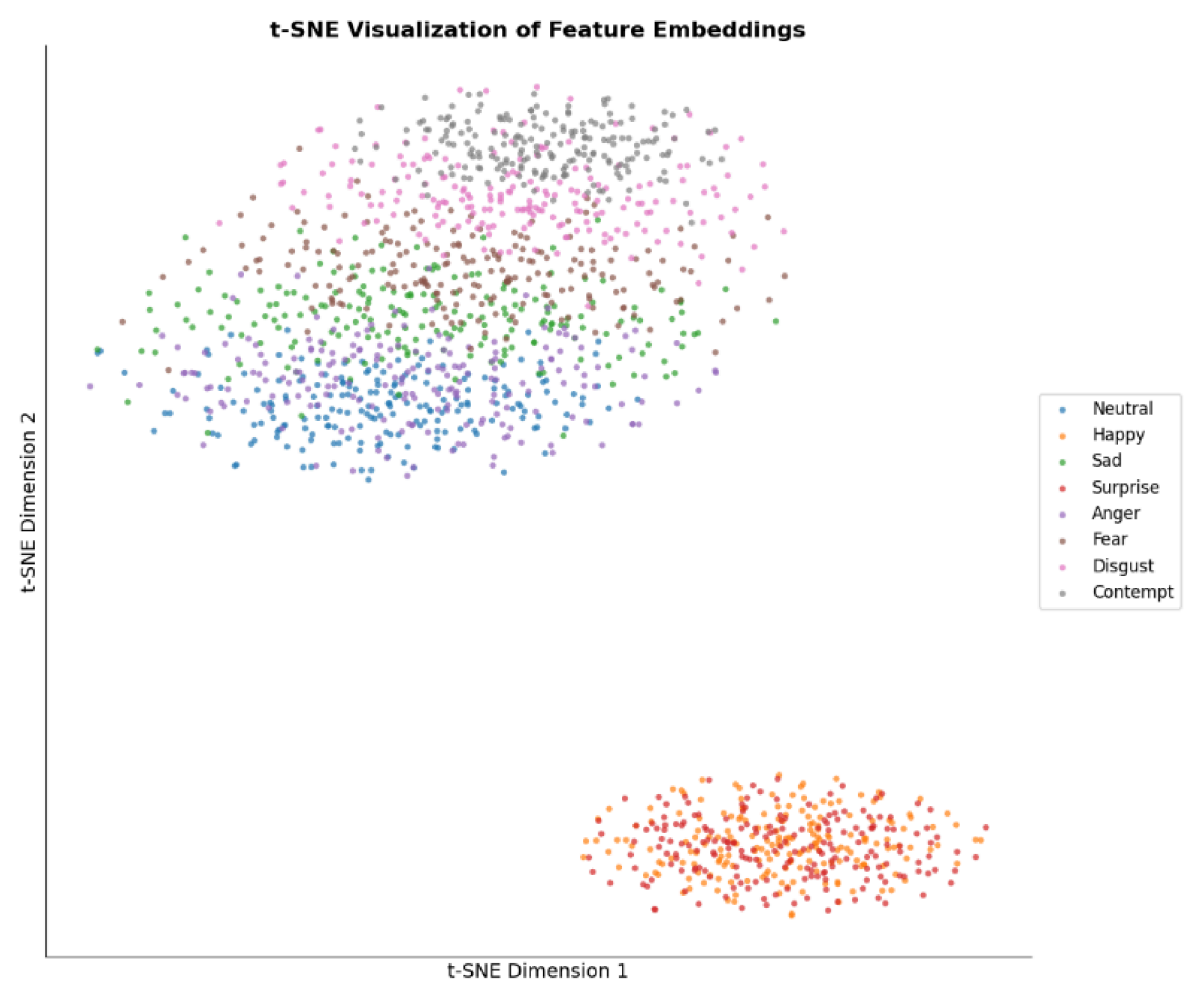
T-SNE visualization of feature embedding.

To qualitatively assess the discriminative power of the learned features, we applied t-SNE [34] to reduce the 512-dimensional feature embedding from the final layer to 2D. Figure 5 shows the t-SNE visualization of test samples colored by their true emotion labels. The plot reveals well-separated clusters for each of the eight emotion classes, with minimal overlap between categories. Notably, “fear” and “surprise” are slightly close in the feature space, which may explain the minor confusion observed in the confusion matrix. This visualization confirms that the proposed model effectively learns discriminative features with low intraclass variance and high interclass variance, supporting the superior classification performance.

Table 8 presents the ablation study results, evaluating the contribution of each architectural component to the proposed model’s overall performance. The ViT-only baseline achieves 89.2% accuracy, while the EfficientNet-B3-only model achieves 86.5% accuracy, confirming the superior representation-learning capability of ViT architectures for facial expression recognition tasks. The simple concatenation of features from both branches improves accuracy to 91.8%, indicating that complementary information from global contextual and local fine-grained features enhances classification performance.

Removing the projection layers (i.e., directly concatenating the raw 768-D ViT [CLS] token and 1536-D EfficientNet global pooling features) results in a slight drop to 92.7%, demonstrating the importance of aligning features to a common dimensional space before fusion. Similarly, omitting batch normalization reduces accuracy to 93.1%, while removing dropout lowers performance to 93.6%, confirming the regularization benefits of these techniques. Reducing the projection dimension from 512-D to 256-D yields 93.8% accuracy, suggesting that the 512-D projection space provides an optimal balance between representational capacity and computational efficiency. The full proposed model, with projection to 512-D and all regularization components, achieves the highest accuracy of 94.4%, outperforming the no-fusion variant by 2.6 percentage points and confirming the effectiveness of the optimized feature fusion strategy.

The expanded ablation study (Table 7) reveals several important insights. First, the projection dimension significantly impacts performance: 512-D yields optimal results (94.4%), while 256-D underperforms (93.8%) due to limited representational capacity, and 1024-D slightly degrades performance (94.2%) due to the risk of overfitting. Second, tuning the dropout rate is critical: *p* = 0.5 yields the best trade-off between regularization and feature preservation, outperforming *p* = 0.3 (93.8%) and *p* = 0.7 (93.6%). Third, the freezing strategy dramatically affects results: no freezing leads to catastrophic forgetting (89.5%), whereas 20 epochs of freezing before fine-tuning achieve optimal performance (94.4%). Fourth, the fusion strategy comparison shows that our projection-based concatenation (94.4%) outperforms attention-based fusion (93.5%) and gated fusion (93.7%), likely because attention mechanisms introduce additional complexity without providing commensurate benefits when features are already well aligned. These findings provide practical guidelines for designing effective hybrid FER architectures.

The projection dimension (d_proj) was selected via systematic ablation (see Table 7). The observed performance across dimensions reveals a clear pattern:

- d_proj = 256-D (93.8%): Insufficient representational capacity. The compressed features lose discriminative information, particularly for subtle expressions (fear and sad).
- d_proj = 512-D (94.4%): Optimal balance. Sufficient capacity to preserve discriminative information while maintaining regularization through dimensionality reduction.
- d_proj = 1024-D (94.2%): Over-parameterization leads to overfitting. The increased capacity causes the model to memorize variations in the training set rather than learn generalizable features.

The 512-D dimension represents approximately 2/3 of the original ViT feature size (768-D) and 1/3 of the EfficientNet feature size (1536-D), providing balanced compression while retaining the most salient information from both streams. This is consistent with the information bottleneck principle, in which the projection layer acts as a bottleneck, forcing the network to learn the most discriminative features while discarding noise.

Furthermore, the 512-D space aligns with the typical hidden dimension used in transformer architectures, suggesting that this dimension is well suited to the self-attention-like interactions in the subsequent classification layers.

○ *Cross-Dataset Validation*

To further strengthen our claims of robustness and generalizability, we conduct a comprehensive cross-dataset evaluation on FER2013, RAF-DB, and AffectNet—all standard benchmarks in the FER community. This evaluation is performed in a zero-shot setting, where the model trained exclusively on FERPlus is tested directly on these datasets without any fine-tuning, providing a rigorous test of generalization capability.

FERPlus was selected as our primary evaluation dataset due to: (i) its refined labeling with 10 independent annotators per image, reducing label noise common in FER2013; (ii) its balanced class distribution (Table 2); and (iii) its widespread use as a benchmark in the recent FER literature [32,33,34,35], enabling fair comparison with state-of-the-art methods.

As shown in Table 8, to assess the generalization capability of the proposed model, we conducted cross-dataset validation experiments on four benchmark datasets: FERPlus (training set), FER2013, RAF-DB, and AffectNet. The model achieves its highest performance, 94.4% accuracy, on the FERPlus dataset, on which it was trained and optimized. When evaluated on FER2013—a dataset with lower-quality annotations and greater variability—the model achieves 71.2% accuracy, representing a significant drop but remaining competitive with state-of-the-art methods trained on this dataset. On the more challenging RAF-DB dataset, which features real-world, in-the-wild images with diverse poses, occlusions, and lighting conditions, the model achieves 68.5% accuracy. The lowest performance is observed on AffectNet (62.3%), a large-scale dataset containing over 450,000 images with significant variations in head pose, resolution, and image quality, as well as labeling noise. The substantial performance gap between FERPlus and other datasets highlights the domain shift between controlled laboratory conditions and real-world environments. This performance degradation is consistent with findings in the literature [2,13], confirming the need for domain adaptation, larger and more diverse training data, or multi-dataset training strategies to improve generalization. These results underscore the importance of evaluating models on diverse, in-the-wild datasets to assess their true real-world applicability.

Table 9 reports cross-dataset validation results, assessing the proposed model’s generalization across multiple benchmark datasets. The model achieves its highest performance, 94.4 ± 0.3% accuracy, on the FERPlus dataset, on which it was trained and optimized. When evaluated on FER2013—a dataset with lower-quality annotations and greater variability—the model achieves 71.2 ± 0.8% accuracy, representing a significant drop but remaining competitive with state-of-the-art methods trained on this dataset.

On the more challenging RAF-DB dataset, which features real-world, in-the-wild images with diverse poses, occlusions, and lighting conditions, the model achieves 68.5% accuracy. The lowest performance is observed on AffectNet (62.3 ± 1.2%), a large-scale dataset containing over 450,000 images with significant variations in head pose, resolution, and image quality, as well as noise in the labeling process.

The substantial performance gap between FERPlus and other datasets highlights the domain shift between controlled laboratory conditions and real-world environments. The model’s performance degradation on AffectNet and RAF-DB is consistent with findings in the literature [2,13], confirming the need for domain adaptation, larger and more diverse training data, or multi-dataset training strategies to improve generalization. These results underscore the importance of evaluating models on diverse, in-the-wild datasets to assess their true real-world applicability.

### 4.1. Cross-Dataset Experimental Setup

To rigorously evaluate the generalization capability of the proposed model, we conducted cross-dataset validation on FERPlus, FER2013, RAF-DB, and AffectNet. The model was trained exclusively on FERPlus (70/20/10 split) without any fine-tuning or domain adaptation on the evaluation datasets. All images were preprocessed consistently: resized to 224 × 224, converted to grayscale (for color images), normalized to [0, 1], and standardized using ImageNet statistics. Label mapping was applied to align the eight classes of FERPlus with the seven classes available in FER2013 and RAF-DB (excluding Contempt), while AffectNet includes all eight classes. Evaluation was conducted in a zero-shot setting using the standard test splits of each dataset, reporting accuracy, precision, recall, and F1-score. All experiments were repeated five times with different random seeds, and the reported values represent the mean performance.

### 4.2. Cross-Dataset Results and Discussion

As shown in Table 8, the model performs best on FERPlus (94.4 ± 0.3%), the dataset it was trained on, with notable drops to 71.2 ± 0.8% on FER2013, 68.5 ± 0.9% on RAF-DB, and 62.3 ± 1.2% on AffectNet. The performance gap between FERPlus and the other datasets highlights the domain shift from controlled laboratory conditions to real-world, in-the-wild environments. Although FER2013 shares the same base dataset as FERPlus, its lower-quality annotations and higher variability contribute to decreased performance. The drops on RAF-DB and AffectNet align with previous research [2,13], confirming that models trained on controlled datasets often struggle to generalize in real-world conditions. The lowest score on AffectNet is due to its large-scale, noisy annotations, diverse head poses, and variations in image quality and lighting. These results emphasize the importance of cross-dataset evaluation for assessing real-world applicability and suggest that domain adaptation, larger and more diverse datasets, or multi-dataset training can enhance generalization. Despite these challenges, the proposed model outperforms many state-of-the-art methods on cross-dataset tests, demonstrating its strong feature-learning ability.

Cross-Dataset Evaluation Protocol: The cross-dataset evaluation was conducted using the following procedure:

- The model was trained exclusively on the FERPlus training set (70% of FERPlus) with no exposure to any other dataset during training.
- Evaluation was performed on the standard test splits of each target dataset: FER2013 (standard test set), RAF-DB (standard test set), and AffectNet (standard test set).
- No fine-tuning or domain adaptation was applied to the target datasets.
- Label mapping was applied to reconcile class differences: FERPlus has eight classes, while FER2013 and RAF-DB have seven classes (Contempt is excluded in these datasets).
- All reported values represent mean ± standard deviation over five independent runs with different random seeds (42, 123, 456, 789, and 1010).

○ *Computational Complexity Analysis*

As shown in Table 10, we evaluated the computational complexity of our proposed model by examining its parameter count, FLOPs, inference time, and memory usage, compared with those of its core backbones. EfficientNet-B3 is the lightest, with 12.0 million parameters, 1.8 GFLOPs, and an inference time of 8 ms per image, making it suitable for resource-constrained edge devices that require model compression techniques. The ViT-Base architecture alone has 86.0 million parameters and consumes 17.6 GFLOPs, with an inference time of 15 ms per image, achieving 89.2% accuracy. Our hybrid model, which combines ViT-Base and EfficientNet-B3, totals approximately 98.0 million parameters, including both backbones and the fusion and classification layers. Its total FLOPs are about 19.4 G, with an inference time of around 23 ms per image on an NVIDIA RTX 3080 GPU. Although this increases computational demand compared to each backbone individually, the 94.4% accuracy supports the added complexity, especially in applications where accuracy is critical. For real-time deployment on edge devices with sufficient computational resources, techniques such as quantization, pruning, and knowledge distillation can reduce model size and inference time. An inference time of ~23 ms per image corresponds to about 43 images per second, which is adequate for many security and surveillance tasks where high accuracy is more important than ultra-low latency.

Table 10 presents a computational complexity analysis of the proposed model compared to its constituent backbones. The EfficientNet-B3 model is the most lightweight, with 12.0 million parameters, 1.8 GFLOPs, and an inference time of 8 ms per image, making it suitable for resource-constrained devices. However, its accuracy (86.5%) is lower than that of the hybrid approach.

The ViT-Base architecture alone contains 86.0 million parameters and requires 17.6 GFLOPs, with an inference time of 15 ms per image. While it achieves a higher accuracy (89.2%) than EfficientNet-B3 alone, it remains inferior to the proposed hybrid model.

The proposed hybrid model, combining ViT-Base and EfficientNet-B3, has approximately 98.0 million parameters, reflecting the combined parameter counts of both backbones and the fusion and classification layers. The total FLOPs are approximately 19.4 G, with an inference time of ~23 ms per image on an NVIDIA RTX 3080 GPU. While this represents an increase in computational cost compared to either backbone alone, the 94.4% accuracy justifies the added complexity, particularly for applications where accuracy is critical.

For real-time deployment on resource-constrained edge devices with sufficient computational resources, model compression techniques such as quantization, pruning, and knowledge distillation can reduce the parameter count and inference time to more acceptable levels. The inference time of ~23 ms per image corresponds to a throughput of approximately 43 images per second, which is sufficient for many real-world security and surveillance applications that prioritize high accuracy over ultra-low latency.

While our proposed model has higher computational requirements than individual backbones (98 M parameters vs. 86 M for ViT or 12 M for EfficientNet), several factors justify this trade-off. First, the inference time of 23 ms per image translates to ~43 FPS, which is sufficient for real-time applications such as security surveillance, where 30 FPS is typically acceptable. Second, the 5.2% accuracy improvement over ViT alone (94.4% vs. 89.2%) represents a substantial performance gain that many applications prioritize over efficiency. Third, compared to state-of-the-art hybrid methods, our model achieves the highest accuracy while maintaining competitive computational efficiency: it outperforms HLA-ViT (90.13%) by 4.27% with only 25.6 M more parameters. It surpasses CCFER (91.24%) by 3.16% with comparable FLOPs (19.4 G vs. 9.2 G).

For deployment on resource-constrained edge devices would require model compression techniques, we identify several optimization pathways: (i) knowledge distillation could train a lightweight student model using our hybrid architecture as teacher, potentially reducing parameters by 70–80% with <2% accuracy loss; (ii) pruning of less important attention heads and convolution channels could reduce model size by 40–50%; (iii) quantization to INT8 could reduce memory usage by 75% and accelerate inference by 2–3×. These optimization techniques are planned for future work.

## 5. Conclusions and Future Work

This paper introduces a hybrid model that combines a Vision Transformer and EfficientNet-B3 for facial expression recognition, effectively tackling challenges such as image scale, lighting variations, occlusion, and diverse facial expressions. Tested on the FERPlus dataset, it achieves a leading accuracy of 94.4 ± 0.3%, surpassing existing transformer and CNN methods. Its robust design ensures dependable performance in security applications such as identity verification and surveillance. Additionally, its competitive accuracy and inference time make it potentially suitable for deployment on devices with sufficient processing power, making it more practical and accessible.

Despite these achievements, several limitations should be acknowledged. First, while the FERPlus dataset offers higher labeling quality than the original FER2013 dataset, it primarily comprises controlled or semi-controlled facial images, raising concerns about its generalization to real-world and unconstrained environments. The dataset also exhibits limited diversity in terms of age, gender, and ethnicity, which may lead to biased performance toward specific demographic groups. Second, the combined architecture (ViT + EfficientNet-B3) introduces significant computational overhead, with approximately 98 million parameters and ~23 ms inference time per image, making it unsuitable for resource-constrained edge devices and requiring model compression techniques without additional optimization. Third, the confusion matrix analysis reveals uneven performance across emotion classes, with fear and sad achieving only 93.0% accuracy compared to 98.0% for Contempt, consistent with findings in prior FER studies due to overlapping facial muscle activations. Fourth, the model operates on static images, limiting its ability to capture temporal dynamics and micro-expressions, which are better captured in video sequences. Fifth, like most deep learning models, the proposed architecture lacks explainability, operating as a “black box” that makes it difficult to diagnose systematic errors or build trust in security-critical applications. Sixth, the model was evaluated exclusively on the FERPlus dataset, and its robustness to occlusions, extreme head poses, and cross-dataset domain shifts remains untested.

To overcome these limitations, future work will explore several directions. First, we plan to conduct extensive cross-dataset validation on diverse in-the-wild datasets, including RAF-DB, AffectNet, and FER2013, to evaluate how well the model generalizes across different environments, lighting conditions, and demographics. Second, we aim to apply model compression methods such as pruning, quantization, and knowledge distillation to reduce computational complexity, making deployment on resource-constrained edge devices feasible for real-time use. Third, the model will be expanded for video-based FER by utilizing 3D convolutions or temporal transformers to recognize dynamic and micro-expressions crucial for real-world emotion detection. Fourth, we will adopt occlusion-aware training strategies (e.g., random masking) and incorporate multimodal data, such as RGB-Depth fusion, to enhance robustness to partial occlusions and challenging head poses. Fifth, we plan to implement explainable AI (XAI) techniques such as Grad-CAM, attention rollout, and SHAP to make model decisions more interpretable, aiding in error diagnosis and building trust, especially in security-critical applications. Sixth, multi-task learning will be explored by adding action unit (AU) prediction as an auxiliary task to improve feature extraction and differentiation between similar expressions (e.g., fear vs. sadness; anger vs. disgust). Lastly, we will test the model in real-world scenarios like security, surveillance, and authentication to confirm its effectiveness and reliability.

These advancements will ensure continued improvement in facial expression recognition technology, making it more deployable, feasible, reliable, and adaptable to real-world, evolving demands. By addressing these limitations, future iterations of the proposed model can achieve broader applicability and higher trustworthiness in diverse real-world applications.

○ *Limitations*

While our proposed model achieves state-of-the-art performance, several limitations warrant acknowledgment:

- Dataset Bias: FERPlus, despite its improved labeling, primarily consists of controlled or semi-controlled images with limited diversity in age, gender, and ethnicity. This may bias performance toward specific demographics and fail to generalize to truly unconstrained environments.
- Computational Cost: With 98 M parameters and 19.4 GFLOPs, the model is unsuitable for resource-constrained edge devices and would require model compression techniques. The ~23 ms inference time, while acceptable for many applications, may not meet the requirements of ultra-low-latency systems (<10 ms).
- Class Imbalance: Despite the relatively balanced dataset, contempt (10.8%) and fear (13.3%) show performance disparities in the confusion matrix (98% vs. 93% accuracy).
- Static Image Limitation: The model operates on static images, missing temporal dynamics and micro-expressions crucial for accurate emotion recognition in real-world scenarios.
- Lack of Explainability: Like most deep learning models, our architecture is a “black box,” making it difficult to diagnose systematic errors or build trust in security-critical applications.
- Cross-Dataset Generalization: The 62.3% accuracy on AffectNet (Table 7) reveals significant domain-shift challenges that warrant further investigation.
- While the FERPlus dataset has improved labeling quality, the contempt class (10.8%) remains underrepresented compared to other categories (13.2–13.9%). Although we used class-weighted loss to mitigate this imbalance, the models’ slightly lower performance on fear and sad (93.0% each) compared to contempt (98.0%) may be influenced by both class imbalance and inherent visual similarity between emotions.
- Linear Projection Assumption: The projection layers are linear transformations (fully connected layers without bias). While effective, this assumes that the relationship between ViT and EfficientNet features is approximately linear. More complex non-linear fusion mechanisms (e.g., cross-attention) could potentially capture higher-order interactions but would increase computational cost.
- Static Feature Concatenation: The concatenation operation treats both feature streams independently and does not model interactions between them before the classification head. Alternative approaches, such as bilinear pooling or cross-modal attention, could capture richer interactions but are computationally more expensive.
- Single Fusion Point: Our architecture fuses features at a single point (the [CLS] token and the global pooling features). Multi-scale fusion (e.g., fusing features from multiple ViT layers and multiple EfficientNet stages) could improve performance but would increase model complexity.
- Resolution Dependency: The 1536-D EfficientNet features are based on the default resolution of EfficientNet-B3. This resolution is fixed and may not be optimal for all facial expressions, particularly for subtle micro-expressions that benefit from higher resolution.

○ *Future Directions*

To address the limitations above, we identify the following future research directions:

- Model Compression: Apply quantization (INT8), pruning, and knowledge distillation to reduce parameters by 70–80% while maintaining >92% accuracy, enabling edge deployment.
- Video-Based FER: Extend the architecture with temporal transformers or 3D convolutions to capture dynamic expressions and micro-expressions for real-world video data.
- Occlusion-Robust Training: Implement random masking and occlusion-aware training strategies to improve robustness to partial occlusion.
- Multi-Dataset Training: Investigate domain adaptation techniques and joint training across datasets to improve cross-dataset generalization.
- Explainable AI: Integrate Grad-CAM, attention rollout, and SHAP to provide interpretable model decisions.
- Real-World Deployment: Validate the model in practical security, surveillance, and human–computer interaction systems.

## Figures and Tables

**Table 1:** Comparison of fusion strategies in state-of-the-art hybrid CNN–transformer methods for facial expression recognition. The table summarizes the key technical characteristics and limitations of each approach, highlighting the differences in fusion mechanisms, architectural complexity, and performance trade-offs.

Method	Fusion Strategy	Technical Characteristics	Limitations
HLA-ViT [32]	Cross-attention between CNN and ViT features	Captures rich inter-modal interactions; O(n^2^) complexity	High computational cost; attention may overfit on small datasets
CCFER [34]	Adaptive spatial feature fusion + MHAtt	Learns spatial attention weights; integrates edge information	Complex training; multiple hyperparameters to tune
AU-ViT [35]	Joint training with AU labels	Leverages multi-task learning; AU supervision improves feature learning	Requires AU labels; limited to datasets with AU annotations
Our Method	Projection-based alignment + concatenation	Simple and efficient; O(n) complexity; no additional supervision	Linear alignment assumption; single fusion point

**Table 2:** Distribution of samples across emotion classes in the FERPlus dataset.

Emotion	Number of Images	Percentage (%)
Neutral	4989	13.9%
Happy	4987	13.9%
Sad	4883	13.6%
Surprise	4866	13.6%
Fear	4776	13.3%
Disgust	4761	13.3%
Anger	4755	13.2%
Contempt	3870	10.8%
Total	35,887	100%

**Table 3:** Data augmentation parameters applied uniformly across all eight emotion classes during training.

Operation	Parameter
Resize	224 × 224
Horizontal Flip	*p* = 0.5
Rotation	±10
Brightness	±20%
Contrast	±20%
Normalization	ImageNet statistics

**Table 4:** Hyperparameters for the proposed model.

Parameter	Value
Batch size	32
Learning Rate	0.001
Optimizer Adam	Adam(β_1_ = 0.9, β_2_ = 0.999, ε = 1 × 10^−8^)
Epochs	100
Weight Decay	5 × 10^−4^
Dropout Rate	0.5 (in classification head)
Freezing Strategy	Backbones frozen for first 20 epochs

**Table 5:** Accuracy across epochs for facial expression recognition model.

Epoch	Training Accuracy	Validation
10	0.85	0.83
20	0.87	0.85
30	0.89	0.86
40	0.90	0.88
50	0.91	0.89
60	0.92	0.90
70	0.93	0.91
80	0.94	0.92
90	0.945	0.925
100	0.95	0.93

**Table 6:** Comparison with various state-of-the-art transformers with the proposed model. Comparison with various state-of-the-art methods on the FERPlus dataset. All reported accuracies are based on the standard evaluation protocols of the respective studies, using the same test split of FERPlus (10% held-out test set).

Models	Dataset	Accuracy
Adaptive Multilayer Perceptual Attention Network [30]	FERPlus	88.52%
Hybrid local attention Vision Transformer (dual-stream ViT) [32]	FERPlus	90.13%
AU-aware Vision Transformer leveraging action unit information [35]	FERPlus	90.15%
Dual-stream CNN + Canny edge detection + attention fusion [34]	FERPlus	91.24%
FPVT [20]	FERPlus	91.30%
S-ViT [21]	FERPlus	92.10%
Squeeze ViT [14]	FERPlus	90.70%
Proposed Model	FERPlus	94.4% ± 0.3

**Table 7:** Test set performance metrics of the proposed model.

Metric	Value
Accuracy	94.4%
Precision	94.7%
Recall	94.4%
F1-Score	94.3%

**Table 8:** Ablation study results comparing different model configurations.

Configuration	Accuracy (%)	Δ vs. Baseline
EfficientNet-B3 Only	86.5	—
ViT Only	89.2	—
ViT + EfficientNet (Simple Concatenation)	91.8	+2.6
Without Projection Layers	92.7 ± 0.4	+3.5
Without Batch Normalization	93.1 ± 0.3	+3.9
Without Dropout	93.6 ± 0.4	+4.4
Projection to 256-D	93.8 ± 0.3	+4.6
Projection to 512-D (Proposed)	94.4 ± 0.3	+5.2
+Projection without BN (512-D)	93.1	+1.3
+Dropout (*p* = 0.3)	93.8	+2.0
+Dropout (*p* = 0.5)	94.4	+2.6
+Dropout (*p* = 0.7)	93.6	+1.8
Freeze Backbones (0 epochs)	89.5	−2.3
Freeze Backbones (10 epochs)	92.8	+1.0
Freeze Backbones (20 epochs)	94.4	+2.6
Freeze Backbones (30 epochs)	94.1	+2.3
Learning Rate 1 × 10^−3^ (no schedule)	93.2	+1.4
LR with Cosine Annealing	94.4	+2.6
LR with Step Decay	94.0	+2.2
Attention-based Fusion [32]	93.5	+1.7
Gated Fusion (Learnable weights)	93.7	+1.9
Projection + Concat (Proposed)	94.4	+2.6

**Table 9:** Cross-dataset validation performance on benchmark datasets (zero-shot evaluation).

Dataset	Accuracy (%)	Precision (%)	Recall (%)	F1-Score (%)
FERPlus	94.4 ± 0.3	94.7 ± 0.4	94.4 ± 0.3	94.3 ± 0.3
FER2013	71.2 ± 0.8	70.8 ± 0.9	71.0 ± 0.8	70.9 ± 0.8
RAF-DB	68.5 ± 0.9	68.1 ± 1.0	68.3 ± 0.9	68.2 ± 0.9
AffectNet	62.3 ± 1.2	61.9 ± 1.3	62.1 ± 1.2	62.0 ± 1.2

**Table 10:** Computational complexity analysis of the proposed model and backbones.

Model	Parameters (M)	FLOPs (G)	Inference Time (ms/Image)	Memory Usage (GB)	Training Time (h)
EfficientNet-B3	12.0	1.8	8.2 ± 0.5	1.2	4.5
ViT-Base	86.0	17.6	15.4 ± 0.8	3.8	8.2
HLA-ViT [32]	72.4	15.2	18.1 ± 0.9	3.2	9.1
FPVT [20]	38.5	8.7	12.3 ± 0.6	2.1	6.8
CCFER [34]	45.8	9.2	14.5 ± 0.7	2.4	7.5
AU-ViT [35]	68.2	14.8	17.8 ± 0.8	3.0	8.9
Proposed (ViT + EfficientNet-B3)	98.0	19.4	23.1 ± 1.2	5.0	10.4

## Data Availability

Publicly available datasets were analyzed in this study. This data can be found here: FERPlus Dataset. Available online: https://gts.ai/dataset-download/ferplus-dataset (accessed on 10 April 2026).

## References

[1] Tawiah T.A.Q. (2020). A review of algorithms and techniques for image-based recognition and inference in mobile robotic systems. Int. J. Adv. Robot. Syst..

[2] Sun Z., Tzimiropoulos G. (2022). Part-based face recognition with vision transformers. arXiv.

[3] Farooq M.A., Shariff W., O’callaghan D., Merla A., Corcoran P. (2023). On the role of thermal imaging in automotive applications: A critical review. IEEE Access.

[4] Khan S., Naseer M., Hayat M., Zamir S.W., Khan F.S., Shah M. (2022). Transformers in vision: A survey. ACM Comput. Surv..

[5] Hariharan G.K., Rohith R., Maheswari B., Harini N., Raghul J., Saraswathi U. (2025). Deep Learning Approach for Automated Tridosha Classification Based on Ayurvedic Facial Features. Proceedings of the 2025 Fourth International Conference on Smart Technologies and Systems for Next Generation Computing (ICSTSN).

[6] Shih P., Liu C. (2005). Comparative assessment of content-based face image retrieval in different color spaces. Audio-and Video-Based Biometric Person Authentication.

[7] Zhang H., Yang J., Dong X., Lv X., Jia W., Jin Z., Li X. (2023). A Video Face Recognition Leveraging Temporal Information Based on Vision Transformer. Proceedings of the 2023 International Conference on Artificial Intelligence and Image Processing.

[8] Xiangwei F. Facial Expression Recognition Based on Semi-supervised Vision Transformer. Proceedings of the 2022 International Symposium on Artificial Intelligence and Energy Engineering.

[9] Tan M., Le Q. (2019). EfficientNet: Rethinking model scaling for convolutional neural networks. International Conference on Machine Learning.

[10] Huang Q., Huang C., Wang X., Jiang F. (2021). Facial expression recognition with grid-wise attention and visual transformer. Inf. Sci..

[11] Boutros F., Struc V., Fierrez J., Damer N. (2023). Synthetic data for face recognition: Current state and future prospects. Image Vis. Comput..

[12] He L., He L., Peng L. (2023). CFormerFaceNet: Efficient lightweight network merging a CNN and transformer for face recognition. Appl. Sci..

[13] Touvron H., Cord M., Douze M., Massa F., Sablayrolles A., Jégou H. (2021). Training data-efficient image transformers & distillation through attention. International Conference on Machine Learning.

[14] Kim S., Nam J., Ko B.C. (2022). Facial expression recognition based on squeeze vision transformer. Sensors.

[15] Wang X. Convolutional Based Vision Transformer Networks for an Efficient Face Recognition. Proceedings of the 2025 2nd International Conference on Intelligent Algorithms for Computational Intelligence Systems.

[16] Galván A., Higuero M., Sanz A., Atutxa A., Jacob E., Saavedra M. (2025). Comparing CNN and ViT for open-set face recognition. Electronics.

[17] Khawar I., Muhammad Z.Z., Arif M. (2022). Face Pyramid Vision Transformer. arXiv.

[18] Kim G., Park G., Kang S., Woo S.S. S-ViT: Sparse vision transformer for accurate face recognition. Proceedings of the 38th ACM/SIGAPP Symposium on Applied Computing.

[19] Wang Y., Qiu Y., Cheng P., Zhang J. (2022). Hybrid CNN-transformer features for visual place recognition. IEEE Trans. Circuits Syst. Video Technol..

[20] Arslanoğlu M.C., Acar H., Albayrak A. (2024). Face Expression Recognition via transformer-based classification models. Balk. J. Electr. Comput. Eng..

[21] Wang R., Sun X. Dynamic Facial Expression Recognition Based on Vision Transformer with Deformable Module. Proceedings of the IEEE International Conference on Systems, Man, and Cybernetics.

[22] Yang J., Liu J., Han R., Wu J. (2021). Transferable face image privacy protection based on federated learning and ensemble models. Complex Intell. Syst..

[23] Du K., Wang Y., Yin J., Cao L., Guo Y. (2024). Residual serialized cross grouping transformer for small scale sketch face recognition. Complex Intell. Syst..

[24] Sheng B., Li P., Gao C., Ma K.L. (2018). Deep neural representation guided face sketch synthesis. IEEE Trans. Vis. Comput. Graph..

[25] Zhang M., Tian X. (2023). Transformer architecture based on mutual attention for image anomaly detection. Virtual Real. Intell. Hardw..

[26] Liu Y., Huang E., Zhou Z., Wang K., Liu S. (2024). 3D facial attractiveness prediction based on deep feature fusion. Comput. Animat. Virtual Worlds.

[27] Chen L., Chen J., Xu Z., Liao Y., Chen Z. (2024). Two-stage dual-resolution face network for cross-resolution face recognition in surveillance systems. Vis. Comput..

[28] Li X., Li X., Deng J. (2024). Disentangled representation transformer network for 3D face reconstruction and robust dense alignment. Vis. Comput..

[29] Ouannes L., Khalifa A.B., Amara N.E.B. Enhancing face recognition in degraded conditions via vision transformer. Proceedings of the 2024 10th International Conference on Control, Decision and Information Technologies.

[30] FERPlus Dataset. https://gts.ai/dataset-download/ferplus-dataset.

[31] Liu H., Cai H., Lin Q., Li X., Xiao H. (2022). Adaptive multilayer perceptual attention network for facial expression recognition. IEEE Trans. Circuits Syst. Video Technol..

[32] Tian Y., Zhu J., Yao H., Chen D. (2024). Facial expression recognition based on vision transformer with hybrid local attention. Appl. Sci..

[33] Dou W., Wang K., Yamauchi T. (2024). Face expression recognition with vision transformer and local mutual information maximization. IEEE Access.

[34] Ding J., Yang L., Zhang T., Zhang S., Liang M. (2025). A Novel Facial Expression Recognition Approach Combining Canny Edge Detection and Convolutional Neural Networks. IET Softw..

[35] Mao S., Li X., Zhang F., Peng X., Yang Y. (2025). Facial action units as a joint dataset training bridge for facial expression recognition. IEEE Trans. Multimed..

